# Transcriptomic features of tumour-infiltrating CD4^low^CD8^high^ double positive αβ T cells in melanoma

**DOI:** 10.1038/s41598-020-62664-x

**Published:** 2020-04-03

**Authors:** Tiphaine Parrot, Romain Oger, Mathilde Allard, Juliette Desfrançois, Diane Raingeard de la Blétière, Anne Coutolleau, Laurence Preisser, Amir Khammari, Brigitte Dréno, Yves Delneste, Philippe Guardiola, Delphine Fradin, Nadine Gervois

**Affiliations:** 1 Université de Nantes, Inserm, CRCINA, F-44000 Nantes, France; 2 LabEx IGO, Université de Nantes, Nantes, France; 3 Cytometry Facility «CytoCell», Federative Structure Research François Bonamy, Nantes, France; 4 Onco-hematological genomics service, Centre Hospitalier Universitaire, Angers, France; 5 Université d’Angers, Inserm, CRCINA, F-44000 Nantes, France; 6 Université de Nantes, CHU Nantes, Inserm, CRCINA, F-44000 Nantes, France

**Keywords:** Skin cancer, Transcriptomics, Immunology, T cells

## Abstract

Peripheral CD4^+^CD8^+^ double positive (DP) T cells are a phenotypically and functionally heterogeneous population depending on their origin and pathologic context. We previously identified among tumour infiltrating lymphocytes in melanoma, a tumour-reactive MHC class-I restricted CD4^low^CD8^high^ DP αβ T-cell subpopulation with CD4-like function. In this study, we used an in-depth comparative transriptomic analysis of intra-melanoma DP T cells and CD4 and CD8 single positive (SP) T cells, to better comprehend the origin of this DP phenotype, and define the transcriptomic signature of activated DP T cells. We observed that intra-melanoma DP T cells were transcriptome-wise closer to their CD8 SP T-cell counterparts in terms of number of genes differentially expressed (97 in common with CD8 SP T cells and 15 with CD4 SP T cells) but presented hallmarks of a transition to a CD4-like functional profile (*CD40LG*) with a decreased cytotoxic signature (*KLRC1*) in favour of an increased cytokine-receptor interaction signature (*IL4, IL24, IL17A…*). This unleashed CD4-like program could be the results of the observed unbalanced expression of the THPOK/Runx3 transcription factors in DP T cells. Overall, this study allow us to speculate that intra-melanoma DP T cells arise from CD8 SP T cells being reprogrammed to a helper function.

## Introduction

Contrary to the CD4^+^CD8^+^ double positive (DP) thymocytes, well described as a T cell development stage, peripheral DP αβ T cells had attracted less attention mostly because of their weak frequency in the peripheral blood of healthy human donors (1 to 3%)^1,2^. Nonetheless, following diverse inflammatory processes from viral or parasitic infections^3–5^, to autoimmune diseases^6,7^ or cancer^8–14^, this subpopulation can become quite predominant in the blood or in the inflamed tissue/organ. This suggests a causal relationship between DP T cell emergence and disease. From these studies, DP T cells appeared as a more heterogeneous population than initially thought. Based on the co-expression level of the CD8 and CD4 co-receptors, peripheral DP T cells can be subdivided into three major subtypes: (i) the CD4^high^CD8^low^ phenotype expressing the CD8αα homodimer, (ii) the CD4^high^CD8^high^ phenotype and (iii) the CD4^low^CD8^high^ phenotype both expressing the CD8αβ heterodimer^15,16^. Although not always clearly demonstrated, these diverse phenotypes are presumably resulting from diverse origins. Thus, it has been proposed that CD4^high^CD8^low^ DP T cells derive from peripheral CD4 T cells that following activation with the appropriate co-stimuli such as IL-4, TGF-β or retinoic acid can induce the CD8αα co-receptor expression at a low level^17^. On the other hand, the CD4^low^CD8^high^ phenotype is considered to be emerging from peripheral CD8 T cells that after activation with strong stimuli can reexpress the CD4 co-receptor at a low level^18,19^. Regarding the CD4^high^CD8^high^ phenotype, less is known and either a thymus or a peripheral origin can be considered.

In our previous work, we have described the enrichment in the tumour infiltrate of several solid cancers including melanoma, of a class-I-restricted DP T-cell population presenting the CD4^low^CD8^high^ phenotype^9,10^. This population was not increased in the peripheral blood suggesting the influence of the tumour microenvironment upon the development of DP T cells. Because of their class-I restriction and their CD4^low^CD8^high^ phenotype, intra-melanoma DP T cells are more likely to derive from intra-melanoma CD8 T cells. On the other hand, we demonstrated that these cells were poorly cytolytic and shared instead functional similarities with CD4 T cells^20^.

To further understand the ontogeny of DP T cells and the molecular mechanisms behind this mixed phenotype, we conducted a transcriptome analysis comparing activated intra-melanoma DP T cells to conventional CD4 and CD8 single positive (SP) αβ T cells from 8 melanoma patients. Even though DP T cells and CD8 SP T cells were functionally different, we showed that they shared a similar Vβ repertoire and were transcription-wise closer to each other than to the CD4 SP population. This study suggests that intra-melanoma DP T cells could be the result of a functional plasticity of CD8 T cells following activation and exposure to the tumour microenvironment.

## Results

### Side by side comparison of the Vβ repertoire diversity between intra-melanoma CD4^low^CD8^high^ DP, SP CD4 and SP CD8 T cells

To investigate the hypothesis of a CD8-derived origin of intra-melanoma DP T cells, we first conducted a paired comparison of their T Cell Receptor (TCR) Vβ repertoire with single positive CD4 and CD8 tumour infiltrating lymphocytes (TIL). CD4^low^CD8^high^ DP (subsequently appointed DP), CD4 SP and CD8 SP T cell subpopulations were isolated by FACS sorting (Supplementary Fig. S1) from 8 TILs previously established and amplified *in vitro* from melanoma-invaded lymph nodes^10^. The frequencies of DP T cells varied among these individual TILs with a range of 1% (M298) to 13% (M288) (Table 1). In most cases, TILs comprised a higher proportion of CD4 SP T cells compared with CD8 SP T cells. After a step of *in vitro* expansion, the purity of the FACS-sorted sub-populations was validated and accepted for further analysis when above 95% otherwise a second cell sorting was carried out (Supplementary Table S1).

**Table 1:** Distribution of CD3^+^ T cell subsets based on CD4 and CD8 expression by flow cytometry in eight melanoma TILs.

	M125	M265	M288	M291	M298	M305	M314	M329	Mean +/− SEM
**% DP**	3	2	13	10	1	3	10	2	5.5 +/− 1.7
**% CD4 SP**	86	35	52	3	73	34	71	73	53.4 +/− 9.8
**% CD8 SP**	10	54	31	74	12	62	17	16	34.5 +/− 8.9
**% DN**	1	9	4	13	14	1	2	9	6.6 +/− 1.9

**Figure 1:**
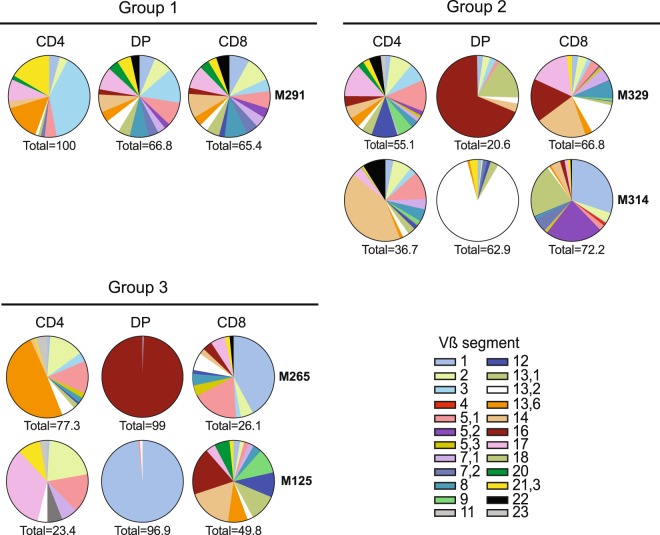
TCR Vβ repertoire distribution between FACS-sorted and expanded intra-melanoma DP, CD4 SP and CD8 SP T cells from TILs patients. Pie charts representing the frequency of expression of each Vβ segment determined by flow cytometry using a panel of 24 anti-Vβ antibodies. The total percentage indicates the overall repertoire covered by these antibodies.

The TCR Vβ repertoire was compared using a panel of antibodies directed against 24 known Vβs covering up to 70% of the human TCR Vβ repertoire (Fig. 1). Out of these 8 populations, 3 were not included in the comparison because of a Vβ coverage below 20% (M288, M298 and M305). On the 5 remaining populations, we observed three different repertoire patterns of DP T cells: (i) a polyclonal and diversified repertoire very similar to that of CD8 SP T cells (M291); (ii) an oligoclonal repertoire less diversified than SP T cell compartments marked by the prevalence of some Vβ (M314 and M329); (iii) a restricted repertoire with a predominant Vβ, suggesting the amplification of a rather clonal population (95% of Vβ1 for M125 and 98% of Vβ16 for M265). As we described before on a smaller number of samples^10^, despite the strong dominance of DP T-cell populations expressing one particular Vβ chain (1, 13.2 or 16), there was no clear recurrence of a particular Vβ usage. For the subgroup 1 the strong overlap of the TCR Vβ repertoire between DP T cells and CD8 SP T cells suggests a CD8-derived origin of the DP T-cell population. This overlapping is not as clear for the others groups once the DP T cell repertoire is less diverse, nevertheless CD8 SP T cells express the predominant DP Vβ TCR.

### Comparison of the activated transcriptomic profile of intra-melanoma DP with SP CD4 and CD8 T cells

**Figure 2:**
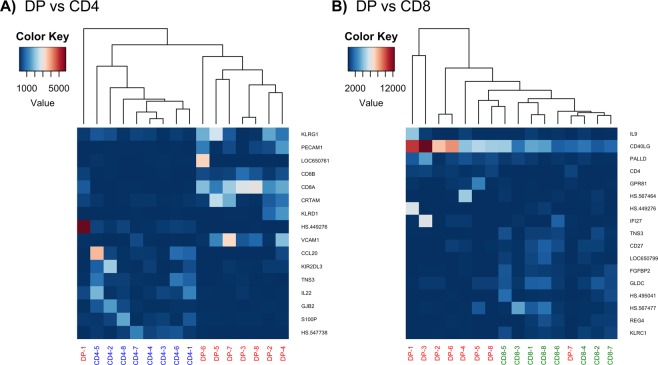
Differentially expressed genes (DEGs) between activated DP and SP CD4 and CD8 T cells. Heatmap and unsupervised hierarchical clustering of the most significantly DEGs between activated DP and (**A**) CD4 SP T cells or (**B**) CD8 SP T cells, matching the following criteria: p < 0.05 by analysis of variance, −1.5 ≤ mean log2 fold change ≥ 1.5. A color bar with scales for each heatmap is included, from dark red to dark blue indicative of high and low normalized expression value respectively. This figure was created using the heatmap.2 package implemented in gplots v3.0.3 in R (https://www.rdocumentation.org/packages/gplots/versions/3.0.3/topics/heatmap.2).

We previously attributed a co-stimulatory function of intra-melanoma DP T cells through the engagement of the CD40L pathway^20^. Here, we sought to define in more details the specific transcriptomic signature of intra-melanoma DP T cells following unspecific activation and possibly identify new functions, using a microarray-based technology (HumanHT-12 v3 Expression BeadChip, Illumina). The analysis was performed on purified bulk CD4, CD8 SP and DP T cells from TILs of 8 melanoma patients left stimulated for 6 hours with plate-bound anti-CD3. Overall, DP T cells were surprisingly very similar to both CD4 and CD8 SP T cells with only 16 and 17 differentially expressed genes respectively (mean log2 fold change <−1.5 and >1.5, and adjusted p-value ≤ 0.05) (Fig. 2). As an internal control, we observed an increased expression of the *CD8* (α and β) and *CD4* genes by DP T cells compared to CD4 and CD8 SP T cells respectively.

Among the 9 genes more expressed by DP T cells than CD4 SP T cells, we found genes belonging to the killer cell lectin-like receptor (KLR) family (i.e. *KLRG1*, KLR subfamily G member 1, and *KLRD1*, KLR subfamily D member 1, coding for CD94), genes involved in cell adhesion (*VCAM1*, Vascular Cell Adhesion Molecule 1, and *PECAM1*, Platelet Endothelial Cell Adhesion Molecule 1) and CD4^+^ CTL differentiation (*CRTAM*, Class I-Restricted T cell-Associated Molecule) (Fig. 2A). Furthermore, DP T cells were distinct from CD4 SP T cells by their significant lower expression of the IL-22 gene and the NK cell inhibitory receptors gene *KIR2DL3* (Killer Cell Immunoglobulin-Like Receptor, 2 Domains Long Cytoplasmic Tail 3), as well as by their reduced expression of genes involved in cell motility (i.e *GJB2*, Gap Junction Beta-2 protein; *TNS3*, tensin 3; *S100P*, 100 Calcium Binding Protein P) and immune cell recruitment (*CCL20*, Chemokine (C-C motif) Ligand 20).

Compared to CD8 SP T cells, we mainly observed in DP T cells a higher expression of the *CD40LG* and P*ALLD* (paladin) genes and a slight reduced expression of *KLRC1* (KLR subfamily C member) coding for the inhibitor receptor NKG2A, *GLDC* (Glycin Decarboxylase), *CD27*, *FGFBP2* (Fibroblast Growth Factor Binding Protein 2) and *REG4* (REGenerating family member 4) genes (Fig. 2B). As previously observed with CD4 SP T cells, DP T cells showed a reduced expression of *TNS3* compared to CD8 SP T cells as well, highlighting this gene as specific feature of DP T cells.

### Biological functions and pathways enriched in activated intra-melanoma DP T cells

Taking into account the fact that p-value adjustment reduces type I error (false positive) at the expense of increasing the chance of type II error (false negative), we then used relaxed statistical conditions to identify more gene expression differences.

Using unadjusted p-values < 0.05, activated intra-melanoma DP and CD4 T cells showed 562 differentially expressed probes (Supplementary Table S2). An extended Gene Set Enrichment Analysis (GSEA) of these genes revealed enrichment in into 7 pathways mainly involved in T-cell signalling pathways and apoptosis (Table 2). Compared to CD4 T cells, DP T cells were overexpressing genes involved in TCR signalling (NFKB1, MAP3K14, PPP3CA, CD8a, CD8b, IL4, NFAT5) (Supplementary Fig. S2) and/or MAPK signalling (PRKX, MYC, FGFR3, RPS6KA3, GADD45B, DUSP4, NR4A1) or apoptosis (BCL2L1, BIRC3, CASP7). Conversely, DP T cells showed decreased expression of genes participating in cytokine receptor interaction (CSF2RA, IL8, IL6R, OSM, IL21, TNFRSF4, TNFRSF18) (Supplementary Fig. S3).

**Table 2:** Kyoto Encyclopedia of Genes and Genomes (KEGG) pathway enrichment analysis for differentially expressed genes between activated intra-melanoma DP and CD4 SP T cells.

Gene Expression Data Set	NG	P-value	FDR	Genes
KEGG_CYTOKINE_CYTOKINE_RECEPTOR_INTERACTION	13	1.1E-6	2.1E-4	IL4, CSF2RA, IL8, IL6R, OSM, IL21, IL21R, XCL1, TNFRSF13C, TNFSF4, TNFRSF4, TNFSF14, TNFRSF18
KEGG_T_CELL_RECEPTOR_SIGNALING_PATHWAY	8	6.5E-6	4.1E-4	IL4, NFKB1, MAP3K14, PPP3CA, NFAT5, CD8a, CD8b, CD247
KEGG_MAPK_SIGNALING_PATHWAY	12	6.6E-6	4.1E-4	NFKB1, MAP3K14, PPP3CA, MYC, FGFR3, FGF4, PRKX, RPS6KA3, NTRK2, GADD45B, DUSP4, NR4A1
KEGG_JAK_STAT_SIGNALING_PATHWAY	9	1.3E-5	4.7E-4	IL4, CSF2RA, IL6R, OSM, IL21R, IL21, MYC, BCL2L1, SPRY1
KEGG_APOPTOSIS	7	1.5E-5	4.8E-4	NFKB1, MAP3K14, PPP3CA, PRKX, BCL2L1, BIRC3, CASP7
KEGG_CHEMOKINE_SIGNALING_PATHWAY	8	3.5E-4	8.2E-3	IL8, XCL1, NFKB1, PRKX, ADCY1, PRKCD, SHC4, ELMO1
KEGG_WNT_SIGNALING_PATHWAY	7	4.6E-4	9.6E-3	PPP3C4, NFAT5, MYC, PRKX, DVL1, FZS3, AXIN2

NG: number of Genes, FDR: False Discovery Rate.

Intra-melanoma DP T cells were closer to intra-melanoma CD8 T cells with the differential expression of 398 probes (Supplementary Table S3). The assessment for enrichment of gene ontologies revealed enrichment in pathways involved in T-cell activation and signalling, NK cell cytotoxicity and cell adhesion (Table 3). The overexpressed genes by DP T cells compared to CD8 T cells were involved in T-cell receptor signalling (CD40LG, IL4, LCK, CD4) (Supplementary Fig. S4) and cytokine-cytokine receptor interaction (CD40LG, CD30L, IL4, IL24, IL27A, LIF, CCL19, IL17RB, IL31RA) (Supplementary Fig. S5), while the down-regulated genes were more associated to cytotoxicity (CD8b, KLRC1, KLRD1, KLRC2, KLRC3) and cell adhesion (CD8b, VCAM1, CD226, ALCAM).

**Table 3:** Kyoto Encyclopedia of Genes and Genomes (KEGG) pathway enrichment analysis for differentially expressed genes between activated intra-melanoma DP and CD8 SP T cells.

Gene Expression Data Set	NG	P-value	FDR	Genes
KEGG_CYTOKINE_CYTOKINE_RECEPTOR_INTERACTION	11	2.7E-6	5.1E-4	CD40LG, IL4, IL24, LIF, CCL19, IL17A, IL17RB, TNFSF4, TNFRDF18, CD27, TNFSF8
KEGG_T_CELL_RECEPTOR_SIGNALING_PATHWAY	7	1.1E-5	9.5E-4	CD40LG, IL4, CD8b, LCK, NFAT5, NFKBIE
KEGG_ANTIGEN_PROCESSING_AND_PRESENTATION	6	3.6E-6	2.2E-3	CD4, CD8b, KLRC1, KLRD1, KLRC2, KLRC3
KEGG_NATURAL_KILLER_CELL_MEDIATED_CYTOTOXICITY	7	4.8E-5	2.2E-3	LCK, NFAT5, KLRC1, KLRD1, KLRC2, KLRC3, SYK
KEGG_PRIMARY_IMMUNODEFICIENCY	4	9.6E-5	3.6E-3	CD40LG, CD4, CD8b, LCK
KEGG_P53_SIGNALING_PATHWAY	5	1.1E-4	3.5E-3	SERPINE1, CDK6, RFWD2, PMAIP1, TP53AIP1
KEGG_CELL_ADHESION_MOLECULES_CAMS	6	3.4E-4	9.1E-3	CD40LG, CD4, CD8b, VCAM1, CD226, ALCAM

NG: number of Genes, FDR: False Discovery Rate.

To validate the RNA microarray data, expression of 7 genes and two housekeeping genes was analysed by real-time RT-PCR. For most of them, real-time RT-qPCR results (Supplementary Fig. 6) are consistent with those from microarray, even if they are not significant.

### Transcription factor profile of activated intra-melanoma DP T cells

**Figure 3:**
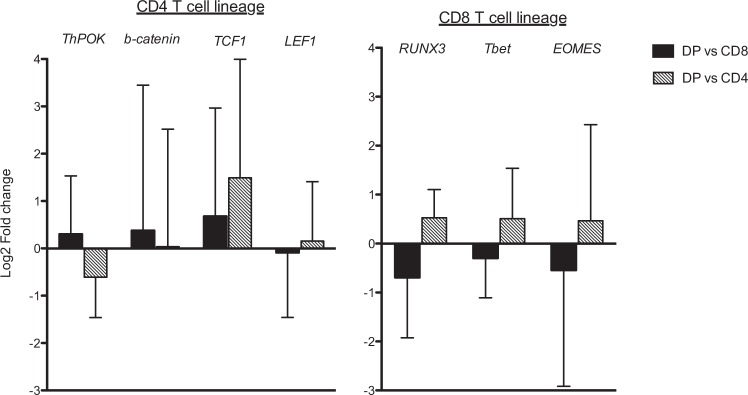
Comparative expression profile of genes encoding transcription factors in intra-melanoma DP. Data were normalized with *RPLP0* and *PPIA* expression using ΔΔCt method and presented as the mean log2 fold ratio difference between DP and CD4 or CD8 SP T cells. The error bars are the standard deviation calculated for mean log2 fold change.

To understand the mechanisms behind DP T cell phenotype and function, we decided to investigate by qPCR the expression of several transcription factors known to be involved in T cell function and CD8 or CD4 T-cell lineage specification in the thymus and/or control in the periphery. Although the inter-donor heterogeneity could not allow us to draw any clear conclusion, we could notice an intermediate expression profile of transcription factors by DP T cells. While DP T cells expressed a lower level of the CD4 T-cell lineage factor ThPOK compared to CD4 SP T cells, they overexpressed it in comparison to SP CD8 T cells. Conversely, RUNX 3 a transcription factor involved in CD8 lineage specification is down regulated in DP T cells compared to CD8 T cells while up regulated in comparison to CD4 T cells. In accordance with our *in vitro* functional data, DP T cells have a reduced expression of T-bet and EOMES involved in cytotoxic function compared to SP CD8 T cells (Fig. 3). In our restrictive set of transcription factor studies, TCF1 appeared to be overexpressed by DP T cells compared to both CD4 and CD8 T cells.

## Discussion

Peripheral DP T cells encompass a highly heterogeneous population based on the expression level of the CD4 and CD8 co-receptors, the hypothesized origin, the described functionality and the inflammatory context studied. We previously documented that this DP TIL sub-population accounts for an average of 6% of total intra-melanoma T cells and may reach up to 13% in some patients. In this study, we aim at extending our current understanding of intra-melanoma CD4^low^CD8^high^ DP T cells by performing an in-depth analysis of their transcriptome profile following activation in comparison to conventional CD4 and CD8 SP T cells.

We first questioned the ontogeny of intra-melanoma DP T cells. Their CD4^low^CD8^high^ phenotype and the HLA class-I restriction are in favour of a CD8-derived origin. To evaluate this hypothesis, we analysed the Vβ repertoire diversity of DP T cells in comparison to their CD4 and CD8 counterparts. We documented that intra-melanoma DP T cells expressed a diverse TCR repertoire without any recurrence of a particular Vβ usage and were mono or pauciclonal in the majority of melanomas, reminiscent to what has been observed for intra-tumour DP T cells in renal cell carcinoma^14^. In most patients, the Vβ repertoire of DP T cells was overlapping with the CD8 T-cell compartment in accordance with Nadler’s group which showed by TCR sequencing a shared clonal representation between DP and CD8 SP T cells on a limited set of donors.

Similarly, transcriptome analysis showed that intra-melanoma DP T cells appeared to be more similar to CD8 SP T cells that to CD4 SP T cells both in terms of genes differentially modulated during TCR activation or differentially expressed after activation. Nonetheless, in agreement with our previous work, DP T cells appeared functionally distinct with a reduced cytotoxic signature and an enriched cytokine-receptor interaction profile shared with CD4 SP T cells. The principal transcript distinguishing DP T cells from CD8 SP T cells was encoding CD40L. We previously documented *in vitro* that, like SP CD4 T cells, and through CD40L involvement, DP T cells were able to induce both proliferation and differentiation of B lymphocytes and maturation of functional DCs able to efficiently prime cytotoxic melanoma-specific CD8 T-cell responses^20^. This CD4-like function was nonetheless not as efficient as CD4 SP T cells in line with the decreased signature in cytokine-receptor interactions by DP T cells compared to CD4 SP T cells observed in this study. Moreover, DP T cells differed from CD4 SP T cells by the expression of CRTAM involved in EOMES induction and cytotoxic T cell (CTL)-like features^21^. We have also compelling arguments to support that DP T cells are terminal differentiated effector T cells^22^ such as the lower expression of *CD27* transcript compared with CD8 SP T cells and the high expression of the NK receptor KLRG1. This last one is thought to be associated with T-cell senescence especially in virus-specific CD8 T cells during persistent antigen stimulation^23,24^. More than a marker for T-cell senescence, signalling through KLRG1 possessing an ITIM in its cytoplasmic domain, may be responsible in part for the defects observed in highly differentiated T cells^25^. Moreover, the high expression of TCF1 on DP T cells could be associated with stemness (high proliferative and self-renewal capacity) as it has been recently described on CD8 T cells in chronic infection^26–28^. In addition, the quantitative higher expression of adhesion molecules (VCAM, CRTAM and PECAM1) in DP T cells in comparison to SP T cells could affect the presence of different immune cell subsets into the microenvironment and regulate tumour immune responses^21,29^. Overall, our results seem to indicate that intra-melanoma DP T cells are derived from SP CD8 T cells transitioning from a cytolytic function to a helper function.

Looking at the transcription factor network known to regulate T-cell specification toward the CD4 or CD8 lineage^30^, we observed an inverse expression pattern of ThPOK and Runx3 in DP T cells in comparison to CD4 SP and CD8 T cells. ThPOK is involved in CD4 T-cell lineage specification in the thymus and is repressed in CD8 T cells^31^. Through its binding to the CD4 silencing element, it prevents the repressive activity of Runx3 allowing CD4 expression^32^, while, by recruiting HDAC on the CD8αβ locus, introduces an epigenetic repression of the CD8 coreceptor in CD4 T cells^33^. ThPOK is also involved in CD4 T-cell functions by repressing cytotoxic genes expression and by inducing helper genes such as CD40L in CD4 T cells^34,35^. The group of Naoe suggests that ThPOK represses CXXC5, which contributes to repressing CD40L expression in CD8 cytotoxic T cells through epigenetic regulation^36^. Thus, the dysregulated expression of ThPOK in DP T cells could explain their phenotype and reprogramming toward the helper lineage. ThPOK expression being regulated by Runx3^37^, its unleashed expression in DP T cells could be the result of the decreased expression level of Runx3 in comparison to CD8 T cells. Runx3 down-regulation by DP T cells compared to CD8 T cells could lead to an inefficient silencing of the CD4 locus allowing the CD4 coexpression at a low level and an inefficient induction of the CD8 lineage-associated genes. As such we observed a slight reduction in EOMES in DP T cells known to be regulated by Runx3 that could explain the inefficient cytolytic of DP T cells^38^.

Interestingly, deregulation of the transcription factors Runx3 and ThPOK has already been described to regulate intestinal CD4 T-cell immunity^39^. It has been shown that CD4 T cells exposed to a microenvironment rich in TGF-β and retinoic acid could acquire Runx3 expression and lost ThPOK expression leading to the emergence of a CD4^high^CD8^low^ phenotype. These experiments provide mechanistic evidence of how transcription factors involved in T-cell lineage choice continue to have a decisive role in cell function in the periphery. We can speculate that the opposite phenomenon, namely the loss of Runx3 and the acquisition of ThPOK, may occur on some CD8^+^ T cells in the tumour microenvironment following sustained activation. This is in accordance with the CD4^low^CD8^high^ phenotype classically recognized as derivative from CD8 T cells reexpressing the CD4 co-receptor following a strong and prolonged activation^18,19^.

Another possible signalling pathway involved in DP T-cell phenotype and helper function could be the Wnt/β-catenin pathway. It has been reported that the induction of β-catenin in peripheral CD8 T cells following activation and/or Wnt signaling^40,41^ leads to the inhibition of the intrinsic HDAC activity of TCF1/LEF1 required for the repression of CD4^+^ T-lineage genes in CD8^+^ T cells such as CD4 and CD40L^42^. Since some Wnt ligands able to induce the β-catenin in DC have been found enriched in melanoma, the same phenomenon could be expected in CD8 T cells. However, in our settings, both the microarray analysis and the qPCR did not revealed any significative differential expression of the β -catenin in CD8 T cells compared to DP T cells.

In conclusion, our study demonstrated that despite their CD4-like functional features, intra-melanoma DP T cells shared a transcriptome profile closer to CD8 T cells suggesting a CD8-derived origin. This is reminiscent with the hypothesis of an antigen-driven expansion and differentiation of CD8 T cells to a DP T cell phenotype in renal cell carcinoma^14^. We can speculate that a prolonged exposure of CD8 SP T cells to antigen and/or inflammation in a peculiar tumour microenvironment could lead to their transcriptomic reprogramming toward CD4-helper like features.

## Methods

### Patients

Tumour infiltrating lymphocytes (TILs) were isolated from metastatic melanoma patients (Unit of Dermato-cancerology, Nantes Hospital, Nantes, France) after signed informed consent (Nantes ethic committee, approval number: 1278197). All experimental protocols were carried out in accordance with relevant guidelines and regulations and, the ethical committee Nantes ethic committee approved this study.

### Purification and amplification of tumour-infiltrating CD4^+^, CD8^+^ SP and DP αβ T cells

TILs were previously obtained from tumour-invaded lymph nodes of eight melanoma patients (M125, M265, M288, M291, M298, M305, M314 and M329). Briefly, TILs were isolated by culturing mechanically disaggregated solid tumor-invaded lymph nodes into 24-well tissue cuture plates in RPMI-1640 medium supplemented with 8% human serum (local production), 150 U/mL human rIL-2 (Proleukin, Novartis), 2 mM L-glutamine, 100 U/mL penicillin and 0.1 mg/mL streptomycin for about 14 days. TILs were then expanded by stimulation with PHA-L (Sigma-Aldrich, L4144) and 150 U/mL human rIL-2 in the presence of allogeneic irradiated feeder cells (PBMCs and B-EBV B cells) in RPMI-1640 medium supplemented with 8% human serum, 2 mM L-glutamine, 100 U/mL penicillin and 0.1 mg/mL streptomycin, as previously described^43^.

Pure CD4^+^, CD8^+^ SP and DP T-cell polyclonal populations were generated by cell sorting in a high purity mode using a BD FACSAria III cell sorter (BD Biosciences). TILs were stained with FITC-conjugated anti-CD3 mAb (Clone SK7, BD Biosciences, 340542), APC-conjugated anti-CD4 mAb (Clone SK3, BD Biosciences, 340672) and BV421-conjugated anti-CD8 mAb (Clone RPA-T8, BD Biosciences, 562428) for 30 min at 4 °C in PBS 0.1% BSA and then washed two times in PBS 0.1% BSA (Sigma-Aldrich, A9576) before cell sorting. Viable TILs were first gated on the basis of their morphology in FSC-A/SSC-A. To ensure that DP T-cell phenotype does not result from doublets of T cells, doublets were excluded using FSC-A/FSC-H and SSC-A/SSC-H dot plots before gating on CD3^+^ T cells and sorting CD4^+^, CD8^+^ SP and DP T-cell subpopulations. To obtain enough cells for the transcriptome analysis, the resulting sorted populations were expanded once as decribed above and their purity was assessed. If the purity was below 95%, the cells were FACS-sorted a second time and the sorted fraction was directly used for the trancriptome analysis.

### Vβ repertoire analysis of melanoma-infiltrating CD4^+^, CD8^+^ SP and DP αβ T cells

Vβ diversity of sorted T-cell sub-populations was determined by flow cytometry using 24 anti-Vβ mAbs included in the IOTest Beta Mark TCR V Kit (Beckman-Coulter, IM3497) and analysed on a FACSCanto II (BD Biosciences).

### RNA extraction, labelling and hybridization

Sorted DP, CD4 and CD8 SP TIL from eight melanoma patients were activated or not for 6 hours by anti-CD3 stimulation (1 µg/mL, local production) and total RNA was extracted from 5 × 10^6^ cells of each subgroup. As we previously described in Parrot *et al*.^20^, after cell lysis with Trizol reagent (Life Technologies, 15596026), total RNA was extracted using the RNeasy Micro kit (Qiagen, 74004) and quantified using a Nanodrop ND-1000 spectrophotometer (Thermo Fisher Scientific, Inc.) according to manufacturer’s recommendations. Integrity of the extracted RNAs was assessed with a Bioanalyzer 2100 using the RNA6000 Nano kit (Agilent Technologies, Inc.) and all samples with a RNA integrity number (RIN) greater or equal to 7.00 were included in our study. The Illumina Total Prep RNA Amplification kit (Ambion, Life Technologies, AMIL1791) was used to generate biotinylated, amplified cRNA from 400 ng of RNA per sample, according to the manufacturer’s recommendations. Hybridization on Illumina HumanHT-12 v3 Expression BeadChips, staining and detection of cRNAs with the I-Scan system were performed in duplicate according to manufacturer’s protocol (Illumina, Inc.). The HumanHT-12 v3 Expression BeadChip contains 48,803 marker probes, of which 27,455 are NM coding transcripts, 7,870 are XM coding transcripts (RefSeq Content, Build 36.2, Release 22), and 12,837 experimentally confirmed mRNA sequences that align to EST clusters (UniGene, Build 199). GenomeStudio 2011 v1 and its Expression Analysis Module (version 1.9.0) were used for signal extraction and quantile normalization (Illumina, Inc.).

### Microarray analysis

The Bioconductor Linear Model for Microarray Analysis (LIMMA) package in R was used^44^ to calculate the differential expression of each probe in the microarray^45,46^. Briefly, in LIMMA, fitting of a linear model to the expression data for each probe is performed and the coefficients obtained describe the design matrix. Instead of simple t-statistics, it provides results for moderated t-statistic, moderated F-statistic, and B-statistic (which demonstrates the log-odds of differential expression), by applying the Empirical Bayes method and shrinking the standard errors towards a common value. Hence, LIMMA produces stable and reproducible results even with a small number of arrays. It also has the advantages of fast computation, simultaneous error rate control across multiple contrasts and genes, and effective prioritizing of results by applying a particular cutoff for fold change. All the original data were deposited in the NCBI’s gene expression Omnibus data base (GSE141465).

### Pathway analysis

All differentially expressed genes were further analysed using the Gene Set Enrichment Analysis (GSEA) method^47^. GSEA is a computational method that determines whether an a priori defined set of genes is associated with different phenotypes or functions. GSEA was carried out by searching Molecular Signature Database (MSigDB) version 4.0 provided by the Broad Institute (http://www.broad.mit.edu/gsea/)^47,48^. The MSigDB gene sets are divided into 8 major collections and we focused in our analysis on the Kyoto Encyclopedia of Genes and Genome (KEGG) gene sets. KEGG pathway database is a collection of pathway maps representing our knowledge on the molecular interaction, reaction and relation networks for numerous processes such as the immune system. All parameters were set to default. Nominal p-values and false discovery rates (FDR) were calculated. A gene set was considered significant with FDR q-value below 0.05.

### Analysis of transcript expression

Total RNA from different TIL sub-populations, prepared as described above, was reverse transcribed using the RevertAid H Minus (ThermoFisher scientific, K1631) or amplified and reverse-transcribed by the whole transcriptome amplification kit (WTA2 Sigma) according to the manufacturer’s protocol. The expressions were then analysed by quantitative PCR. Amplification was done by using Maxima SYBR Green/ROX qPCR Master Mix (ThermoFisher scientific, K0221). Relative gene expression was calculated using the ΔΔCt method normalized against the *RPLP0* and *PPIA* genes (determined as the most stably expressed housekeeping genes in our model). Primer sequences are available on Supplementary Table S4.

### Statistical analyses

Statistical analyses for the quantitative PCR were performed using Prism software v.5 (GraphPad). Correlations were assessed using the Pearson’s test after validating the normal distribution of the data set. P values < 0.05 were considered significant.

## Supplementary information

Supplementary information.

Supplementary information 2.

Supplementary information 3.

Supplementary information 4.

Supplementary information 5.

Supplementary information 6.
