# Supplementary material for: Transcriptomic features of tumour-infiltrating CD4lowCD8high double positive αβ T cells in melanoma
Source: Sci Rep. 2020 Apr 3;10:5900. doi: 10.1038/s41598-020-62664-x (PMC7125144; doi:10.1038/s41598-020-62664-x)
Supplement: Supplementary file 1 — Supplementary information. [file 41598_2020_62664_MOESM1_ESM.docx]

**Supplementary Figures**

**Supplementary Figure S1. CD4, CD8 SP and DP TILs selection.** (A) After gating viable TILs on the basis of their morphology in FSC-A/SSC-A, doublets of cells were excluded using FSC-A vs FSC-H and SSC-A vs SSC-H dot plots before gating on CD3^+^ T cells and sorting CD4^+^, CD8^+^ SP and DP T-cell subpopulations. Representative flow cytometry analysis of distribution of CD3^+^ T cells subsets based on CD4, CD8 in two TIL populations, M125 and M314, before (B) and after two cell sorting (only for DP T cell population) (C). The DP cell percentages are shown in the upper right-hand dial. This figure was created using BD FACSDiva Software version 8.0.2.

**Supplementary Figure S2. T cell receptor signalling pathway analysis of genes differentially expressed between DP and CD4 SP T cells after anti-CD3 activation**. Gene downregulated in DP cells are indicated in green and overexpressed in red and conversely gene unregulated in CD4 SP cells are in green and downregulated in red. KEGG map pathway rendered by Pathview implemented in R3.4.2.

**Supplementary Figure S3. Cytokine-Cytokine receptor interaction pathway analysis of genes differentially expressed between DP and CD4 SP T cells after anti-CD3 activation**. Gene downregulated in DP cells are indicated in green and overexpressed in red and conversely gene upregulated in CD4 SP cells are in green and downregulated in red. KEGG map pathway rendered by Pathview implemented in R3.4.2.

**Supplementary Figure S4. T cell receptor signalling pathway analysis of genes differentially expressed between DP and CD8 SP T cells after anti-CD3 activation.** Gene downregulated in DP cells are indicated in green and overexpressed in red and conversely gene upregulated in CD8 SP cells are in green and downregulated in red. One exception is for the CD4/CD8 box which is in grey, since the CD8 marker is significantly more expressed in CD8^+^ T cells compared to DP T cells, but the CD4 marker is significantly lower in CD8^+^ T cells compared to DP T cells. KEGG map pathway rendered by Pathview implemented in R3.4.2.

**Supplementary Figure S5.** **Cytokine- Cytokine receptor interaction pathway analysis of genes differentially expressed between DP and CD8 SP T cells after anti-CD3 activation**. Gene downregulated in DP cells are indicated in green and overexpressed in red and conversely gene upregulated in CD8 SP cells are in green and downregulated in red. KEGG map pathway rendered by Pathview implemented in R3.4.2.

**Supplementary Figure S6. Validation of transcriptome microarray results by qRT-PCR.** Correlation analysis between data of microarray (x axis) and qRT-PCR (y axis) of 7 differentially expressed genes in DP versus CD4 T cells (A) and in DP versus CD8 T cells (B). Correlation was assessed using the Pearson’s test (P=p.value, R=R squared). This figure was created using Prism 5 for Mac OS X version 5-0c.

**Supplementary Tables**

**Supplementary Table S1. Purity of TIL subpopulations for RNA extraction.**

**Supplementary Table S2. Mean expression values of the differentially expressed genes between DP and CD4 SP T cells.**

**Supplementary Table S3. Mean expression values of the differentially expressed genes between DP and CD8 SP T cells.**

**Supplementary Table S4. Primer sequences used for PCR analysis.**
