# Supplementary material for: Transcriptomic features of tumour-infiltrating CD4lowCD8high double positive αβ T cells in melanoma
Source: Sci Rep. 2020 Apr 3;10:5900. doi: 10.1038/s41598-020-62664-x (PMC7125144; doi:10.1038/s41598-020-62664-x)

Transcriptomic features of tumour-infiltrating CD4^{low}CD8^{high} double positive $\alpha\beta$ T cells in melanoma

Tiphaine Parrot[1,2], Romain Oger[1,2], Mathilde Allard[1,2], Juliette Desfrançois[3], Diane Raingeard de la Blétière[4], Anne Coutolleau[4], Laurence Preisser[5], Amir Khammari[2,6], Brigitte Dréno[2,6], Yves Delneste[5], Philippe Guardiola[4], Delphine Fradin[1,2,¶] and Nadine Gervois[1,2,¶,*].

[1] Université de Nantes, Inserm, CRCINA, F-44000 Nantes, France

[2] LabEx IGO, Université de Nantes, Nantes, France

[3] Cytometry Facility « CytoCell », Federative Structure Research François Bonamy, Nantes, France

[4] Onco-hematological genomics service, Centre Hospitalier Universitaire, Angers, France

[5] Université d'Angers, Inserm, CRCINA, F-44000 Nantes, France

[6] Université de Nantes, CHU Nantes, Inserm, CRCINA, F-44000 Nantes, France

¶ These authors contributed equally to this work.

Supplementary Figure S1

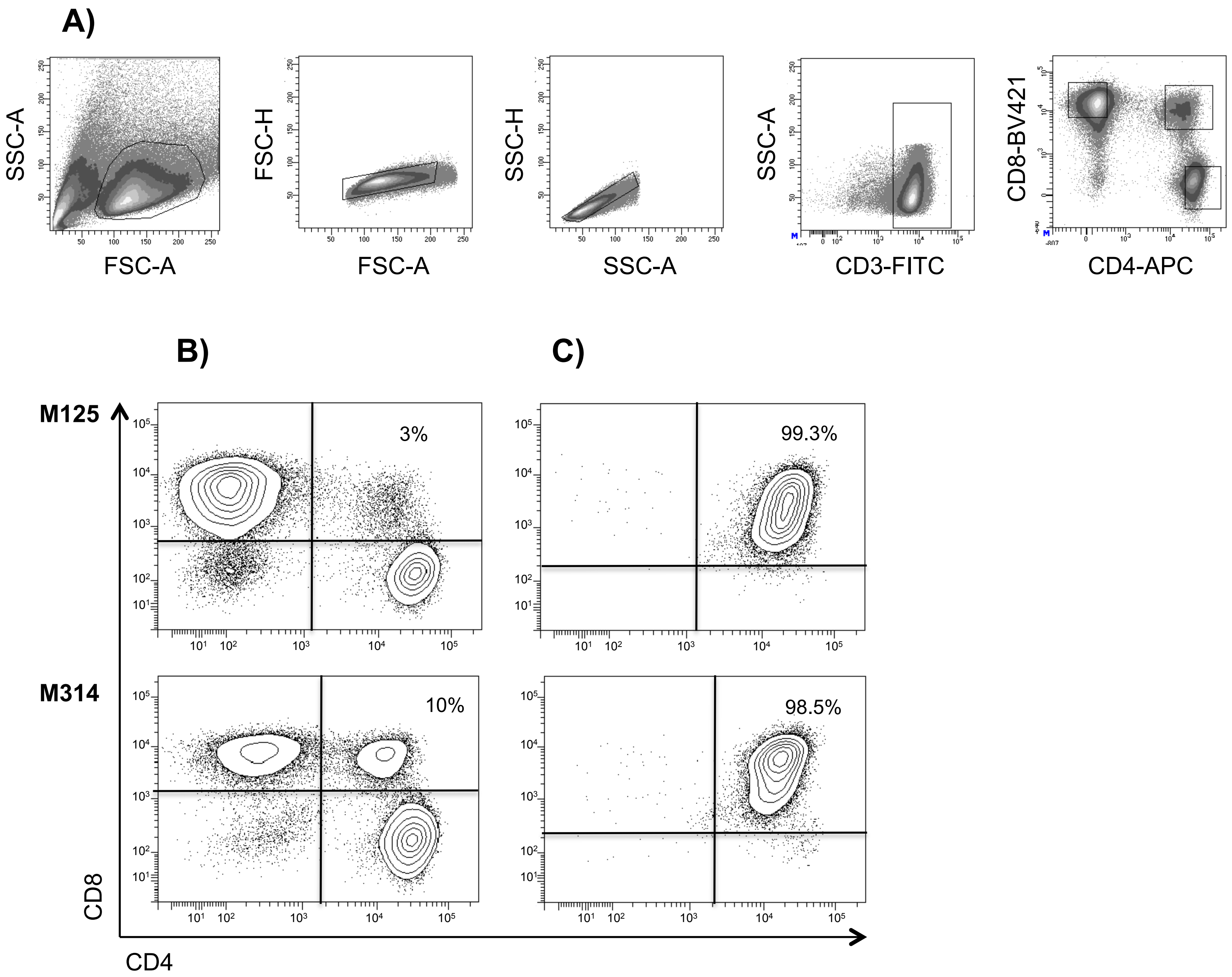

Supplementary Figure S2

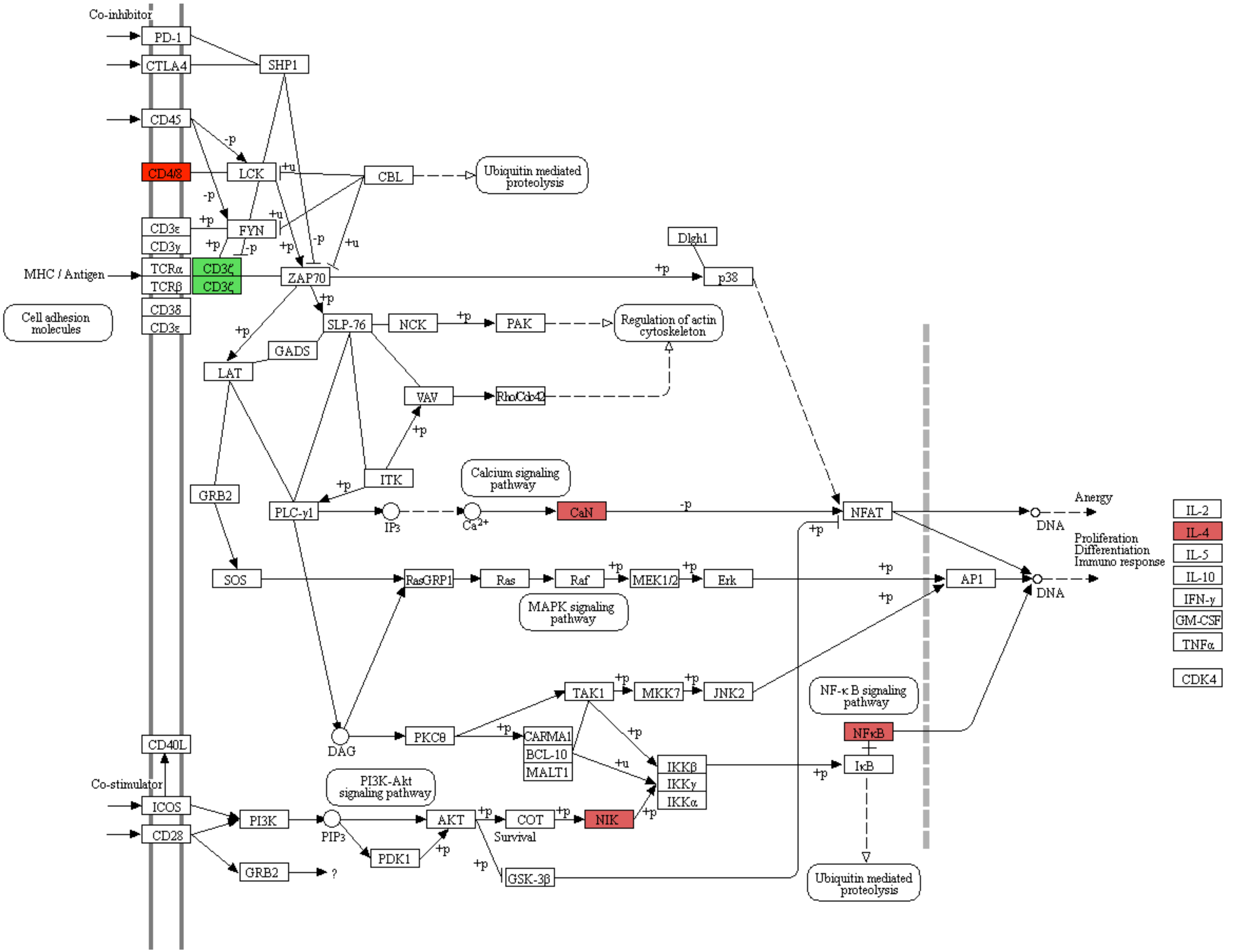

Supplementary Figure S3

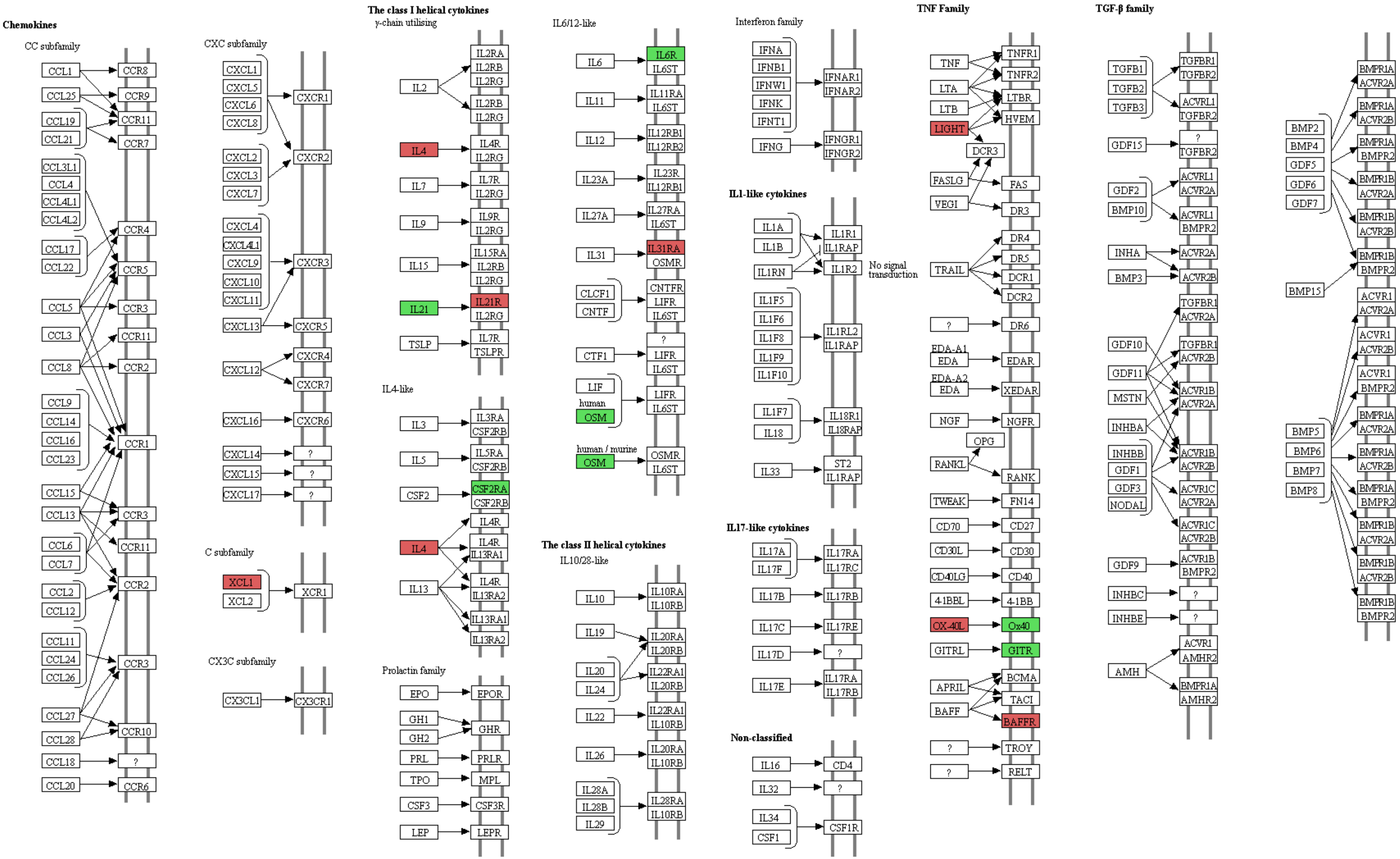

Supplementary Figure S4

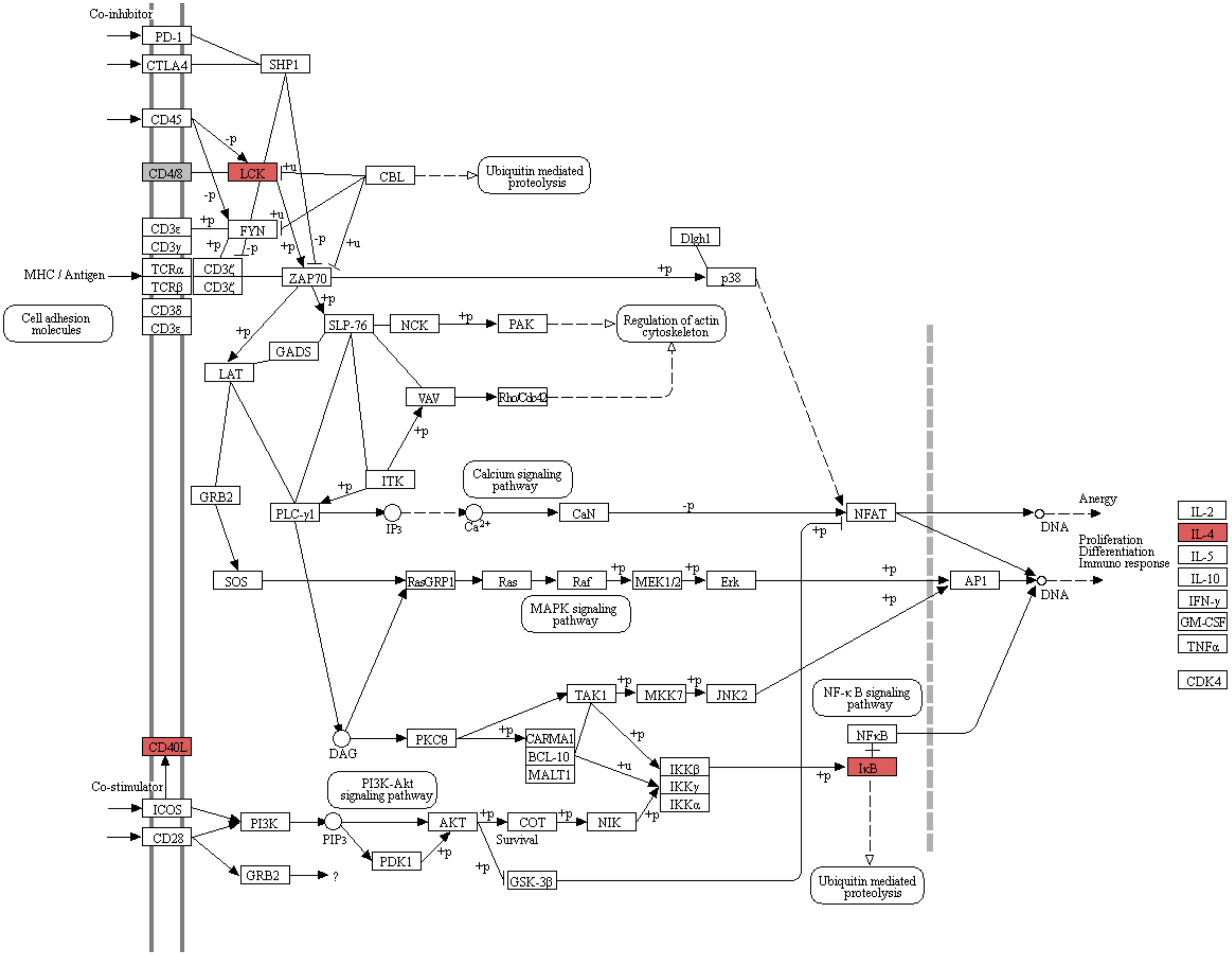

Supplementary Figure S5

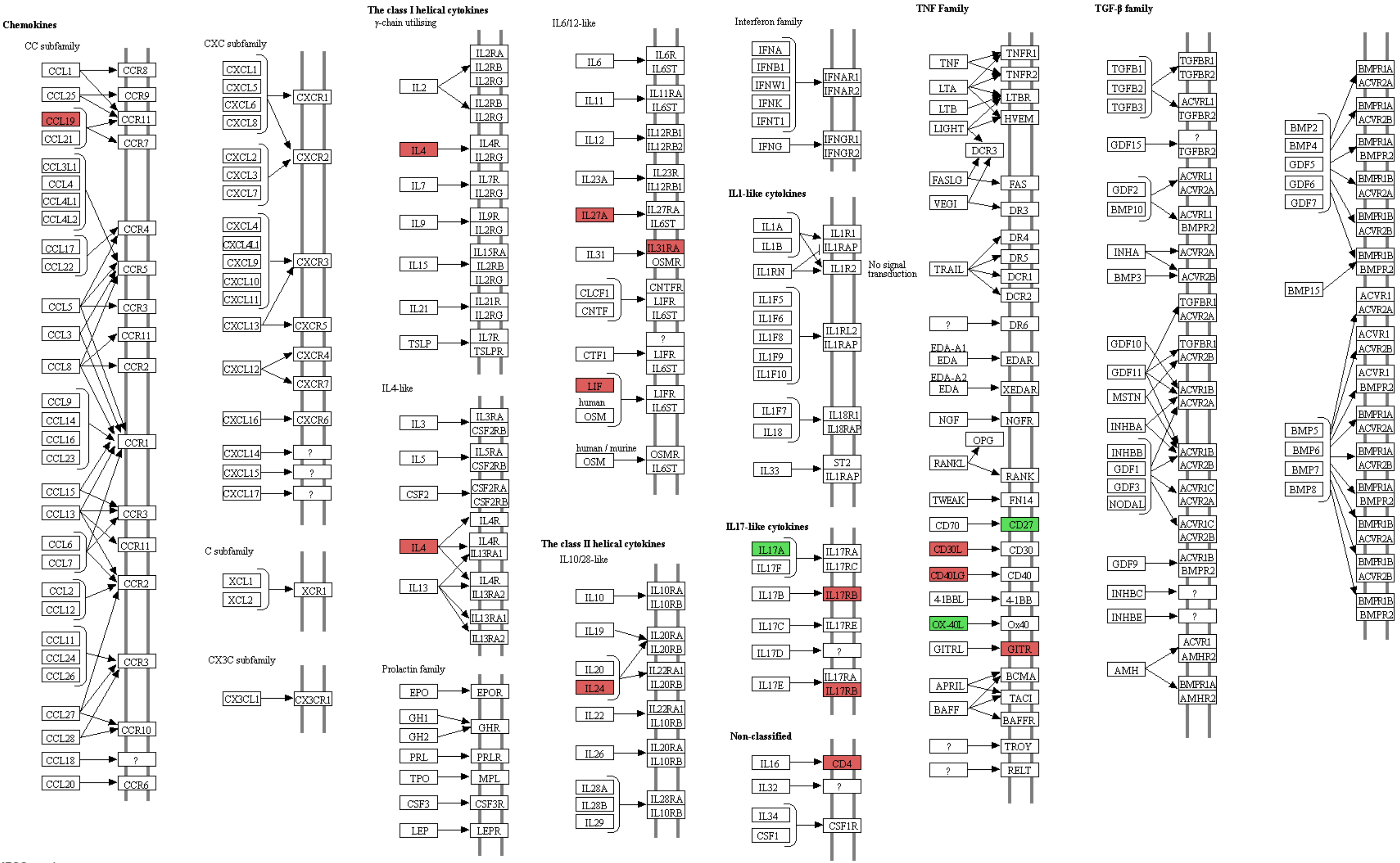

)ata on KEGG graph

Supplementary Figure S6

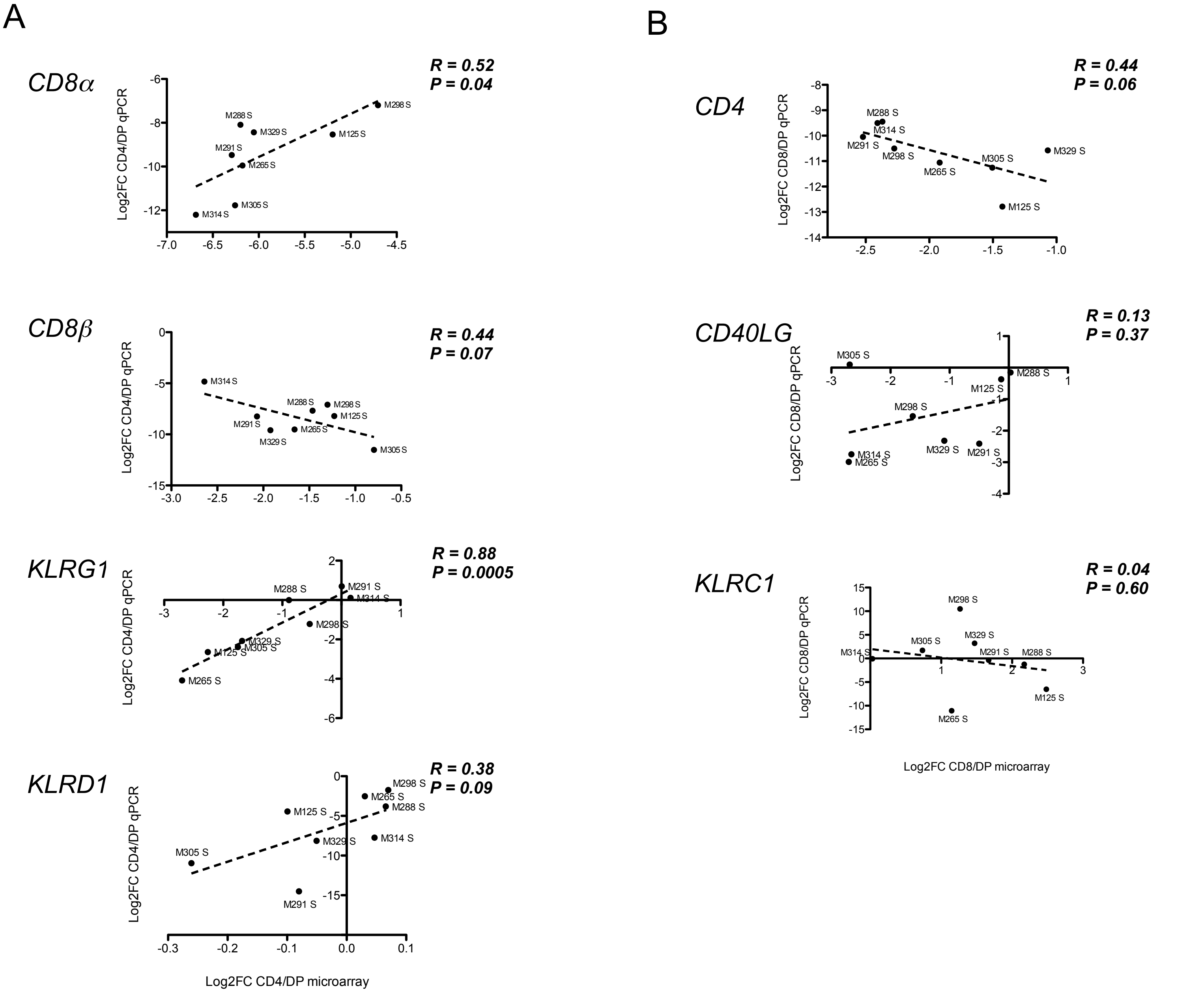

Supplement: Supplementary file 2 — Supplementary information 2. [file 41598_2020_62664_MOESM2_ESM.pdf]
