# Supplementary material for: Transcriptomic features of tumour-infiltrating CD4lowCD8high double positive αβ T cells in melanoma
Source: Sci Rep. 2020 Apr 3;10:5900. doi: 10.1038/s41598-020-62664-x (PMC7125144; doi:10.1038/s41598-020-62664-x)
Supplement: Supplementary file 3 — Supplementary information 3. [file 41598_2020_62664_MOESM3_ESM.pdf]

TIL	CD4		DP		CD8	
	Sort 1 and expansion	Sort 2	Sort 1 and expansion	Sort 2	Sort 1 and expansion	Sort 2
**M125**	95.2%	>98%	81.8%	99.3%	94.1%	>98%
**M265**	92.7%	>98%	73.4%	98.1%	94.3%	>98%
**M288**	96.7%	>98%	87.9%	>98%	97.5%	np
**M291**	99.7%	np	95.8%	>98%	98.7%	np
**M298**	97.6%	np	90.1%	>98%	95.0%	np
**M305**	98.6%	np	95.4%	>98%	96.0%	np
**M314**	97.5%	>98%	52.3%	98.5%	96.3%	>98%
**M329**	98.6%	np	72.2%	99.1%	96.6%	>98%

np: second cell sorting not performed.
