# Supplementary material for: Transcriptomic features of tumour-infiltrating CD4lowCD8high double positive αβ T cells in melanoma
Source: Sci Rep. 2020 Apr 3;10:5900. doi: 10.1038/s41598-020-62664-x (PMC7125144; doi:10.1038/s41598-020-62664-x)
Supplement: Supplementary file 4 — Supplementary information 4. [file 41598_2020_62664_MOESM4_ESM.pdf]

Probe	ILMN_GENE	mean expression in DP	mean expression in CD4
ILMN_1717466	ABCC3	122.0836625	131.7303125
ILMN_1765779	ADAM17	192.6054625	412.3135375
ILMN_2121068	ADAM17	493.46195	167.40635
ILMN_2319326	ADARB1	280.3200375	192.4457375
ILMN_1657442	ADARB1	242.670075	229.044425
ILMN_1742073	ADCY1	115.036275	127.5874375
ILMN_1690352	ADO	2918.596375	2169.35375
ILMN_1703946	ADORA2B	494.462075	330.314275
ILMN_2162799	AHR	377.771775	302.0660125
ILMN_1783818	AKAP11	138.3587125	153.5316625
ILMN_2293758	AKAP11	1000.02265	1215.644825
ILMN_1675507	AKAP2	119.434525	130.259925
ILMN_2401641	ALDH3A2	147.2764875	175.811825
ILMN_1806845	ALG3	352.0554625	279.4771875
ILMN_1684694	ANK1	127.0866125	170.3234875
ILMN_1779882	ANKRD43	191.341525	155.0648125
ILMN_2232478	APOBEC3G	4994.73725	3273.619375
ILMN_1777483	APOOL	165.3081625	186.0063625
ILMN_1808999	ARHGEF19	271.7030625	229.777875
ILMN_1703477	ARHGEF2	5371.471875	4542.0955
ILMN_1741156	ARMCX5	132.0181	169.6418375
ILMN_1654385	ASB13	111.0483375	121.8038125
ILMN_1728055	ASTL	113.5932875	121.4668
ILMN_1799889	ATP6V0D2	134.4231875	123.7101125
ILMN_2089073	ATP9A	193.7456375	386.238875
ILMN_2354334	ATXN2L	171.7579625	152.811625
ILMN_1664177	ATXN7L2	340.03185	271.314975
ILMN_1712159	AVPR1A	110.823025	104.61395125
ILMN_1724480	AXIN2	126.0962875	149.668075
ILMN_1656682	AZIN1	1389.328625	1723.684
ILMN_1711102	B3GNT2	285.5387625	225.8277625
ILMN_1753755	B4GALT2	182.5559875	160.5102625
ILMN_1699727	BAIAP2	142.1661125	131.7139
ILMN_2258749	BAIAP2	148.8311125	129.0781125
ILMN_1742410	BCL2L1	1431.8158375	1076.3801875
ILMN_1766541	BCL2L11	318.1171	411.1526875
ILMN_2359627	BCL2L11	625.5322125	236.926525
ILMN_1776181	BIRC3	13885.431625	11658.94075
ILMN_2099528	BTLA	142.80445	207.613625
ILMN_1778536	BTLA	155.4807625	246.2945125
ILMN_2205963	C10ORF54	1559.3235	1198.6556
ILMN_1719577	C10ORF67	113.0146125	123.4486875
ILMN_2394193	C14ORF138	881.475475	634.2763375
ILMN_1781102	C14ORF138	682.52475	502.8644875
ILMN_1687821	C16ORF45	131.8872	151.139025

ILMN_1737005	C19ORF61	322.4551625	277.09785
ILMN_1681741	C1ORF31	294.3587125	353.7379
ILMN_1742611	C1ORF52	850.069225	739.8390875
ILMN_1764503	C21ORF124	158.360275	143.7822875
ILMN_1690703	C21ORF34	115.46895	123.5114125
ILMN_1716682	C21ORF88	117.3126875	110.945475
ILMN_2182148	C2ORF32	138.31225	124.5262125
ILMN_1760352	C2ORF52	114.1557125	123.002725
ILMN_1815682	C3ORF37	1426.420375	1124.9987
ILMN_1698677	C4ORF27	230.6682875	287.3087125
ILMN_1725276	C4ORF7	103.29797875	113.1434125
ILMN_3248773	C7ORF40	4159.405875	3304.23525
ILMN_1779735	C7ORF59	1235.8656125	1550.430125
ILMN_2082699	C8ORFK36	115.8299	127.6872875
ILMN_1780460	C9ORF41	130.4335375	145.162875
ILMN_2398815	CABP2	123.3663875	115.199825
ILMN_2362974	CASP7	225.6168625	476.9458
ILMN_2373763	CASP7	614.0538	190.3146625
ILMN_1785156	CASRL1	113.772025	122.298025
ILMN_1678061	CASS4	248.3291625	181.170325
ILMN_1655191	CASZ1	562.0506125	394.8632375
ILMN_2302118	CCDC50	1624.9198375	171.32075
ILMN_1738333	CCDC50	146.973925	2920.10875
ILMN_1781769	CCRL1	112.4527375	121.727375
ILMN_2377669	CD247	5826.733375	8455.956125
ILMN_2365307	CD276	131.8805375	178.01945
ILMN_1714602	CD86	338.4163625	692.007375
ILMN_1768482	CD8A	9751.2005	145.39785
ILMN_2353732	CD8A	9029.24125	139.215675
ILMN_1760374	CD8A	1804.8266875	117.3467125
ILMN_1748601	CD8B	431.5438125	121.181175
ILMN_1696675	CES2	1389.2301875	1162.2987375
ILMN_1698144	CFH	109.052025	117.732725
ILMN_1658411	CHD4	2720.273125	997.716
ILMN_1685551	CHD4	1263.092725	2152.8605
ILMN_1677376	CHD7	207.8256375	302.5179875
ILMN_1706397	CHMP6	230.3155	205.80875
ILMN_1678493	CHN1	127.90295	162.5643
ILMN_1663092	CITED2	403.25425	779.670775
ILMN_2086612	CMAH	337.625075	495.0551625
ILMN_1704084	CMAH	392.7680875	615.8342375
ILMN_1679277	CMTM3	131.8978625	120.5936875
ILMN_1696494	CMTM6	2120.25075	3058.721
ILMN_3250201	CNBP	284.8967125	253.24185
ILMN_1666364	COQ10A	443.9298875	364.0340625
ILMN_1653708	CORO1B	272.435325	749.68975

ILMN_3195213	CRCP	135.594375	124.6598
ILMN_1680624	CREG1	397.8826875	766.767025
ILMN_2146418	CRIM1	226.05365	149.219275
ILMN_1796247	CRTAM	835.9947125	167.6586625
ILMN_1782788	CSDA	2558.2949375	1515.9996625
ILMN_2376455	CSF2RA	108.70501	116.7641125
ILMN_1725090	CTHRC1	129.6518	142.4555
ILMN_1651752	CXORF21	128.8182125	150.0818625
ILMN_1670925	CYB5D1	568.099825	429.3799375
ILMN_1704985	CYP27A1	140.0972125	125.0188375
ILMN_1744458	CYP2W1	116.081875	108.764525
ILMN_2173291	CYP4B1	176.569725	261.2261
ILMN_3255061	CYTSB	121.6130125	113.532475
ILMN_1658123	DAZ2	116.0463625	124.7513
ILMN_2174127	DCBLD2	126.5826625	185.1619375
ILMN_1665455	DCUN1D3	880.824675	1167.385825
ILMN_1679405	DDX56	1439.130875	1263.6785125
ILMN_1742276	DKFZP564O0523	249.6219875	220.017275
ILMN_1669972	DKFZP686O24166	192.4335125	159.744475
ILMN_1676128	DNMT3A	288.9854375	229.9106625
ILMN_1763162	DPH2	252.5981	222.5519
ILMN_1679262	DPYSL3	109.77395	118.35125
ILMN_3251672	DSG2	131.659025	156.8512
ILMN_2238302	DST	121.103575	113.3426
ILMN_1730765	DUSP22	323.0078	251.7925875
ILMN_1813275	DUSP22	2119.6825	1676.247125
ILMN_1671809	DUSP22	805.56255	638.727225
ILMN_2283398	DUSP4	244.8520625	604.1105625
ILMN_1808391	DUSP4	849.9330875	182.6198625
ILMN_1657278	DVL1	151.63115	165.688925
ILMN_1708798	EAF2	252.7976	426.068525
ILMN_2347917	EED	836.07285	1090.2042
ILMN_1710150	EED	437.20405	582.1254125
ILMN_1743199	EGR2	7718.59325	5669.9035
ILMN_1784320	ELMO1	569.0353375	200.340225
ILMN_1663627	ELMO1	175.06545	1019.575825
ILMN_1801616	EMP1	240.8997375	426.6521625
ILMN_1784967	EPB41L4B	124.1883375	114.40835
ILMN_1701025	EPHX1	116.1343125	126.6900375
ILMN_1709233	F5	122.0112375	183.568125
ILMN_1695711	FAM105A	362.4441	270.0139
ILMN_1790062	FAM105B	722.2244125	561.6114125
ILMN_2225608	FAM135A	124.7464875	139.2828
ILMN_1691946	FAM173B	187.380025	213.0071125
ILMN_2161285	FAM40B	151.017925	171.8232125
ILMN_3272603	FAM60A	1822.559625	2125.057875

ILMN_1735743	FBLN7	113.42625	145.340075
ILMN_1678404	FBXO11	201.2946125	176.881125
ILMN_2189869	FCF1	177.1207125	161.4144375
ILMN_1810289	FER1L3	135.7501625	200.0892125
ILMN_2381945	FGA	106.50525375	112.9015125
ILMN_1677456	FGF4	109.71295	118.3808
ILMN_1723123	FGFR3	129.685825	118.093275
ILMN_1730325	FLAD1	159.473525	147.6159375
ILMN_1791162	FLJ31945	128.96205	117.62815
ILMN_1699216	FLJ45721	110.3198375	120.67485
ILMN_1698769	FLJ46321	110.3330625	117.7554375
ILMN_2395214	FMNL3	311.0326125	235.9427
ILMN_2235975	FXYD5	398.4085625	314.275275
ILMN_1703408	FZD3	126.02735	159.210025
ILMN_1697559	G6PD	397.35995	694.61935
ILMN_2347949	G6PD	997.4769	283.8295125
ILMN_2151281	GABARAPL1	679.3569	282.9168625
ILMN_1718977	GADD45B	4763.537875	3090.77575
ILMN_1792168	GALE	173.7217375	203.8724625
ILMN_1677092	GEM	426.0250875	249.2017875
ILMN_2367883	GEM	427.0227875	252.8424875
ILMN_3306988	GGTLC1	129.7343875	139.5924625
ILMN_1806754	GLDC	282.2840375	583.6502125
ILMN_1710985	GLIS2	122.6648875	114.957525
ILMN_1680727	GLRX2	327.2719625	424.4057875
ILMN_1789457	GNL1	472.7243375	407.908225
ILMN_2192351	GOLGA8E	132.6267125	120.4236875
ILMN_1766405	GOLM1	138.154425	164.6356375
ILMN_1741224	GPR137C	121.7647	112.24115
ILMN_2122076	GPR149	106.5205625	113.9839
ILMN_1671260	GPR177	111.1332375	122.1913625
ILMN_1705210	GRASP	163.2512	131.8402375
ILMN_1693826	HAVCR2	4491.687875	6328.018375
ILMN_2121408	HBEGF	864.1873	1380.43695
ILMN_1666122	HEG1	1553.2002375	1032.5255875
ILMN_1751596	HIVEP3	222.6949625	185.5715375
ILMN_1686862	HLX	138.7461375	177.6733125
ILMN_2087646	HLX	197.1848625	479.8011
ILMN_1736894	HNRNPA1L2	168.2404375	149.672375
ILMN_2396039	HOXC6	119.0271125	110.5328375
ILMN_1715672	HPCAL1	144.3755	123.0645875
ILMN_1898662	HS,120187	134.868425	126.364525
ILMN_1882112	HS,121525	499.9820375	330.8299125
ILMN_1825443	HS,127352	131.3313	121.5016625
ILMN_1863284	HS,130916	544.1257	688.1707625
ILMN_1822990	HS,131074	131.3671375	119.5193625

ILMN_1893697	HS,162734	124.289625	160.872275
ILMN_1849907	HS,195035	129.98635	119.52105
ILMN_1898124	HS,25892	3379.923375	2764.438
ILMN_1873677	HS,31037	438.4155	271.1116375
ILMN_1867517	HS,37648	160.4479125	259.1760625
ILMN_1882512	HS,406790	545.5332375	402.7753625
ILMN_1819783	HS,415576	162.0745125	196.228075
ILMN_1828584	HS,445843	244.4565375	190.9248125
ILMN_1909784	HS,473425	149.3374375	132.851825
ILMN_1872404	HS,478682	222.2434375	318.071975
ILMN_1836218	HS,481464	290.0757125	238.8275375
ILMN_1823207	HS,504036	130.926375	123.1048125
ILMN_1836958	HS,528873	190.9924125	160.11695
ILMN_1851316	HS,534445	117.1926375	155.6771
ILMN_1914405	HS,535454	113.154225	122.25245
ILMN_1846547	HS,540716	126.2266625	115.9258
ILMN_1859931	HS,543875	137.02375	125.3040125
ILMN_1859863	HS,555181	630.439725	445.552125
ILMN_1849218	HS,559564	163.5716125	142.284725
ILMN_1885147	HS,565650	129.458825	120.7215875
ILMN_1850366	HS,567186	123.7521625	114.7847
ILMN_1895745	HS,571967	115.867825	107.54959625
ILMN_1889135	HS,572124	128.867075	119.7501375
ILMN_1917174	HS,573799	125.0923375	138.583175
ILMN_1907685	HS,578483	128.3556125	117.9920125
ILMN_1857476	HS,578552	138.726	124.4372
ILMN_1814787	ICA1	140.882625	154.3571625
ILMN_2182198	ICT1	813.8803375	954.051
ILMN_1806093	IFRG15	131.268775	122.4219
ILMN_1777449	IFT74	170.0511625	203.8216125
ILMN_1772674	IL21	180.2922625	250.171175
ILMN_1661687	IL21R	169.9773625	151.7439125
ILMN_1786609	IL21R	122.949825	115.0122375
ILMN_1667575	IL31RA	121.9088	113.2774375
ILMN_2389080	IL4	182.2643125	150.111275
ILMN_1754753	IL6R	116.3788625	124.6967
ILMN_2184373	IL8	6877.677875	13171.241625
ILMN_1666733	IL8	2907.2552125	6724.77225
ILMN_2043079	ILDR1	147.1525375	206.1001375
ILMN_1797668	INSM1	146.7948875	176.238
ILMN_1754491	IYD	106.2033375	114.4222375
ILMN_1727438	KCND3	135.8968625	124.904225
ILMN_1766918	KCNK5	794.18565	613.9132125
ILMN_1674488	KCNN1	119.81295	111.0690125
ILMN_1786326	KCTD15	126.2873875	158.3762125
ILMN_1807767	KIAA0182	671.668875	438.7291375

ILMN_1703969	KIAA0251	204.84375	177.7330625
ILMN_1796751	KIAA1274	220.15865	180.0202375
ILMN_1745813	KIAA1279	307.8592125	379.592125
ILMN_1727833	KIF19	110.52415	121.6488
ILMN_1658399	KLRG1	1019.4413125	305.0695
ILMN_1811261	KRIT1	331.8022375	291.0076
ILMN_1770228	KRT34	115.042125	121.912
ILMN_1737653	KRT78	125.298725	116.0876
ILMN_1746517	KYNU	108.76610875	152.2180375
ILMN_1737514	KYNU	128.874125	178.6142125
ILMN_1807491	LAIR2	424.5693	636.0959875
ILMN_1765212	LARP1B	248.93855	220.043025
ILMN_1767448	LHFP	323.3896125	393.669425
ILMN_1665011	LHX3	111.4308875	121.9615
ILMN_3182893	LOC100128163	160.2106375	131.737775
ILMN_3186782	LOC100128653	117.0578375	127.8044
ILMN_3258717	LOC100128728	119.2176125	128.1048375
ILMN_3256478	LOC100129034	280.8823625	180.9763
ILMN_3189463	LOC100129054	113.7131875	122.6200875
ILMN_3269108	LOC100129058	110.7093625	117.5556375
ILMN_3183346	LOC100129466	111.5897125	120.4336125
ILMN_3259185	LOC100129630	144.6052125	132.0435625
ILMN_3263585	LOC100129916	117.3535	125.3794625
ILMN_3272374	LOC100129936	113.7296875	124.6713125
ILMN_3191030	LOC100129960	221.57625	184.0317625
ILMN_3272590	LOC100129975	240.1995	208.1249375
ILMN_3244935	LOC100130308	2866.891875	3372.706
ILMN_3272243	LOC100130798	123.533675	115.664575
ILMN_3256712	LOC100130828	139.3739375	129.0175
ILMN_3176090	LOC100130919	5665.67775	4503.216875
ILMN_3201183	LOC100131542	121.8019	114.909175
ILMN_3293244	LOC100131744	156.0969625	142.9826125
ILMN_3275696	LOC100131940	1470.606125	1876.976875
ILMN_3247979	LOC100132024	130.6599625	118.845425
ILMN_3244841	LOC100132111	176.7612375	219.0427
ILMN_3235732	LOC100133095	127.018375	113.460975
ILMN_3235126	LOC100133558	127.3909	119.0189
ILMN_3246424	LOC100133600	254.9133625	234.374875
ILMN_3287952	LOC100133800	294.88405	363.4544875
ILMN_3249560	LOC100134291	291.0305125	224.6574
ILMN_1667330	LOC113386	760.6516	657.1964
ILMN_1780613	LOC222967	130.2118625	118.1795375
ILMN_3239925	LOC25845	373.87895	314.5509125
ILMN_2083137	LOC284379	120.4981625	110.787725
ILMN_1694327	LOC285176	304.4042875	256.8753875
ILMN_1806387	LOC340598	118.8239875	127.5102625

ILMN_3204890	LOC345041	188.594825	219.4251125
ILMN_1652628	LOC375295	121.6544375	112.4079375
ILMN_3240698	LOC388279	136.8126375	123.0695
ILMN_3205364	LOC400389	116.2256875	126.3337875
ILMN_1708542	LOC402617	130.518225	117.2024
ILMN_1687568	LOC440337	132.3798875	121.319775
ILMN_1706434	LOC440359	546.1680875	357.5020875
ILMN_1683250	LOC440731	156.4145	194.336025
ILMN_3237507	LOC552889	844.8484875	737.2814875
ILMN_1702620	LOC642374	115.0548875	124.2978125
ILMN_1778128	LOC642780	126.654425	117.99575
ILMN_1658495	LOC642895	121.0121	112.6184
ILMN_1756139	LOC643310	623.03435	739.5667125
ILMN_1661306	LOC643433	3740.649125	4432.404375
ILMN_1797634	LOC643937	127.9826875	120.1303125
ILMN_1737543	LOC644005	110.97815	119.1483375
ILMN_1762424	LOC644024	119.9913875	128.584825
ILMN_3202683	LOC644587	109.4774125	118.288575
ILMN_1775053	LOC644838	113.7869625	121.60695
ILMN_1653352	LOC644975	109.8600875	117.7285125
ILMN_1794617	LOC645104	121.4716375	111.136375
ILMN_1691405	LOC645850	121.6853375	112.6184625
ILMN_1756914	LOC645871	124.3668	115.6890875
ILMN_1767236	LOC648103	137.8830625	127.055875
ILMN_1815474	LOC649470	113.7873375	121.13635
ILMN_1659028	LOC649971	120.3456625	111.607075
ILMN_1683664	LOC650369	3648.25325	4330.755375
ILMN_1718094	LOC651324	125.2957875	118.000725
ILMN_1703297	LOC652512	115.041	124.4829375
ILMN_1745028	LOC652635	116.3534	126.9705375
ILMN_1693804	LOC652675	467.7171375	355.8147875
ILMN_1682518	LOC653197	116.3909125	128.526325
ILMN_1705599	LOC653427	117.92915	130.8821875
ILMN_1688294	LOC653820	139.671425	127.0069375
ILMN_1658460	LOC653884	1436.64125	1207.8795
ILMN_3274532	LOC727997	120.227	111.8513125
ILMN_1771048	LOC728153	641.3562625	525.0454875
ILMN_3298549	LOC729251	121.8936125	110.86455
ILMN_3242786	LOC729915	114.04645	120.362225
ILMN_3301168	LOC730052	334.906575	395.3568375
ILMN_1769220	LOC730713	117.1116375	126.363325
ILMN_1660323	LOC731999	137.9867	120.1952875
ILMN_1656257	LOC732275	122.687125	112.3870375
ILMN_2128795	LRIG1	2793.856375	1939.300125
ILMN_2129161	LRRC32	178.6030625	149.34515
ILMN_1773650	LRRN3	6356.757475	3266.5755

ILMN_2048591	LRRN3	4988.5484	2571.79625
ILMN_1723962	LXN	471.2457375	675.4649375
ILMN_1738646	LY6K	114.219275	122.468025
ILMN_2142117	LYPLAL1	423.7703625	533.46465
ILMN_1722206	MAF	207.803075	277.6727375
ILMN_2361714	MAGEA4	116.4471875	125.8846625
ILMN_1789567	MAGED2	223.026875	170.8879125
ILMN_2327860	MAL	1539.434675	1252.176975
ILMN_2320330	MAL	524.6101	3863.609125
ILMN_1724070	MAP3K14	328.46615	267.353775
ILMN_1671437	MAP3K15	112.578875	121.2781375
ILMN_2408908	MAP4K5	210.4881625	183.0839
ILMN_1669321	MATK	303.9434375	1243.219525
ILMN_2319000	MATK	2201.3496875	210.6620125
ILMN_1720595	MDGA1	137.728175	120.88085
ILMN_1815190	METTL1	1350.8030875	1117.3912875
ILMN_3240524	MFSD6	1196.223125	992.3517
ILMN_1690304	MGC26718	120.9025125	139.950725
ILMN_1862180	MGC3032	224.804525	183.7228125
ILMN_1689624	MINA	264.790225	182.0121375
ILMN_2322986	MINA	161.731375	213.2026875
ILMN_1746025	MINA	184.7290625	335.627325
ILMN_3309955	MIR194-2	124.4496125	110.43653
ILMN_3309224	MIR23A	127.051425	119.9055875
ILMN_3308265	MIR302C	190.0701	276.193525
ILMN_3311070	MIR33B	116.766125	130.3604125
ILMN_3309739	MIRLET7D	170.058125	148.2956125
ILMN_1796316	MMP9	252.678125	174.0402625
ILMN_1792910	MNT	957.4453	728.47885
ILMN_2386008	MPZL1	144.7877375	183.6153625
ILMN_1795739	MPZL3	195.6412	159.1501125
ILMN_1754860	MRPS12	299.0245375	250.495275
ILMN_1774028	MTFR1	242.6448125	278.6319625
ILMN_1772521	MTHFD1L	504.1387625	719.292575
ILMN_1652434	MTHFD2L	132.5168875	142.8606
ILMN_2110908	MYC	2059.82575	1493.797625
ILMN_2198413	MYEOV	126.9943125	115.654225
ILMN_3302919	MYOF	139.8645	192.8229125
ILMN_3244960	NACC1	298.5281375	251.4366375
ILMN_1753111	NAMPT	1118.6925375	823.0425125
ILMN_3249172	NCRNA00092	314.860475	222.5761375
ILMN_1738622	NDNL2	342.5675	262.9673625
ILMN_1695020	NEK3	169.5400125	195.6876
ILMN_1660871	NEK6	258.062625	164.60845
ILMN_1672081	NEURL	115.7533	108.4425625
ILMN_1780291	NFAT5	437.2167125	366.3619875

ILMN_2049766	NFE2L3	1145.7780875	1802.023
ILMN_1694325	NFIX	152.4449125	128.7050125
ILMN_1714965	NFKB1	11953.8075	9401.781875
ILMN_2295879	NGDN	200.5351	177.721325
ILMN_1699778	NKIRAS2	152.7832375	138.216225
ILMN_1724304	NLE1	172.4926875	157.7619375
ILMN_1680435	NPEPPS	166.5661875	145.7647125
ILMN_1814221	NPTX1	261.5133375	499.0164375
ILMN_2410145	NR4A1	158.034375	127.1072625
ILMN_2408566	NR4A1	170.667975	142.738325
ILMN_1782305	NR4A2	2954.1926375	1585.2431125
ILMN_2339955	NR4A2	1543.7046625	835.7517375
ILMN_1687392	NRK	155.898675	145.3212375
ILMN_2366719	NSFL1C	281.7045125	238.1827875
ILMN_1756049	NT5DC3	560.0826125	456.7308
ILMN_2357855	NTRK2	527.14225	848.2588375
ILMN_1672606	OAS1	114.7624875	123.0382875
ILMN_1780546	OSM	1919.0358625	3287.470975
ILMN_1723141	OTUD1	165.951075	147.392175
ILMN_1752008	OTUD1	129.5984125	272.2496
ILMN_1708203	OTUD4	839.6271875	702.7493
ILMN_1716335	P2RXL1	113.4564125	123.57095
ILMN_3300313	P4HTM	1115.918525	407.290725
ILMN_2372136	P4HTM	545.550575	833.9584625
ILMN_1672122	P4HTM	265.18555	220.9265
ILMN_2324909	PARD3B	130.0637375	119.0932
ILMN_3243705	PDXDC1	1403.634625	1182.505125
ILMN_1696962	PDZD8	257.4983625	231.601825
ILMN_1690695	PEX11A	121.4422125	134.399225
ILMN_1737416	PGBD3	213.3573375	187.6849625
ILMN_1666819	PHLDB1	213.1802125	179.077325
ILMN_1789781	PIM3	892.4491375	677.7730375
ILMN_1707748	PIM3	3861.314625	3064.3575
ILMN_1736654	PIP5K1B	177.0581125	219.91065
ILMN_1668514	PIP5K1C	1072.2138	503.7831125
ILMN_2052891	PKD2	570.0073375	712.632725
ILMN_1738742	PLAT	204.8490125	326.848075
ILMN_3240611	PLK5P	120.2158125	109.951025
ILMN_1785265	PLS3	123.506625	158.408375
ILMN_2041788	PLS3	112.07755	203.8545875
ILMN_1764239	PMPCA	1550.961	1232.9043125
ILMN_1724081	POTE14	112.84575	120.6813625
ILMN_1811049	POU2AF1	117.782625	161.747075
ILMN_1758085	PPIL5	124.7599375	115.3503875
ILMN_2291152	PPP2R2C	129.377775	120.5705625
ILMN_1670970	PPP3CA	308.9588875	272.5481875

ILMN_1692429	PQBP1	145.312675	132.2571375
ILMN_1798620	PQLC1	3650.196125	2607.084625
ILMN_1740633	PRF1	8197.24575	5053.4155
ILMN_1801105	PRKCD	1949.76575	1488.570875
ILMN_2205050	PRKX	850.9290625	645.04735
ILMN_1797776	PRSS23	155.6725625	251.682625
ILMN_1693397	PSG4	114.641275	122.1705125
ILMN_1743130	PTGFRN	120.7993375	136.09335
ILMN_2077905	PTGFRN	112.0158125	126.2876625
ILMN_1737398	PTPLAD1	1355.4185	1649.839375
ILMN_2374683	PTPN13	107.414705	152.9331
ILMN_2374687	PTPN13	127.0771125	129.8311375
ILMN_1755023	RAD50	584.6421125	446.7391
ILMN_1757384	RAN	1190.83565	949.03935
ILMN_1780756	RBM23	1862.44675	1588.687375
ILMN_2316844	RBPJ	300.0218875	664.8833375
ILMN_1708537	RBPJ	3074.682125	2180.269475
ILMN_1726913	RBPJ	962.5546	245.898075
ILMN_1728218	RBPMS	122.7761125	150.2406375
ILMN_1720124	RCC2	5598.571625	4548.37775
ILMN_2337492	RCE1	253.5464625	225.3151625
ILMN_2222984	RDH14	1362.1026875	1139.099775
ILMN_2124064	REL	667.371575	406.5054375
ILMN_1766085	REL	597.8773125	400.1663375
ILMN_3247761	REXO1L5P	124.2092375	114.0973375
ILMN_3250850	RFESD	129.525775	139.6356625
ILMN_2100458	RFESD	168.839475	200.41815
ILMN_1808226	RGS16	1426.5430875	1082.6072625
ILMN_1731736	RIN3	300.4034875	149.56675
ILMN_2322499	RORA	169.2427875	260.3259375
ILMN_2322498	RORA	290.1640625	631.453175
ILMN_3306270	RPL31P10	126.5845125	138.489175
ILMN_2338785	RPS14	10060.268625	11386.003
ILMN_1806294	RPS6KA3	739.5304375	589.6896125
ILMN_1781656	SCGB1D1	112.6163625	120.7937375
ILMN_2363591	SDCBP	1767.809025	2456.017875
ILMN_2363586	SDCBP	3134.20325	1274.2947125
ILMN_1803997	SDCCAG3	918.4262375	720.3027
ILMN_2380850	SDCCAG3	485.5220375	388.963425
ILMN_2391912	SEC14L1	484.671225	273.6357
ILMN_1732575	SEC14L1	398.6898125	471.173225
ILMN_2285802	SEC14L1	821.1781	244.0695375
ILMN_1742052	SERPINB9	486.899275	385.5909125
ILMN_2205935	SFXN1	2907.644125	1877.73375
ILMN_1811364	SGPP2	382.273725	156.0188375
ILMN_1812062	SGPP2	136.1322	650.205125

ILMN_1807050	SHC4	273.5412625	394.1268
ILMN_1732410	SLC16A9	116.2132125	139.4617625
ILMN_1785405	SLC17A9	147.213075	122.328975
ILMN_1711838	SLC25A24	391.8149375	757.580225
ILMN_1652465	SLC26A4	138.0933375	221.154475
ILMN_1742731	SLC35A2	966.5698	794.211425
ILMN_1814797	SLC35F3	153.7380375	162.062625
ILMN_1675873	SLC35F3	129.8759875	259.11415
ILMN_1704446	SLC6A10P	175.814525	156.2226875
ILMN_3241985	SNORA13	180.7541625	160.3994875
ILMN_3248890	SNORA24	575.2912125	454.2154
ILMN_3248811	SNORA27	165.913075	147.658675
ILMN_2096747	SNORA33	383.2803625	317.4235
ILMN_3241798	SNORA41	410.8905625	331.4668625
ILMN_1730773	SNORA70	1089.344725	911.304275
ILMN_3244154	SNORA84	193.537475	168.1515875
ILMN_1673181	SNORD100	319.6757875	269.4255875
ILMN_1799381	SNORD14A	363.7462875	300.3156125
ILMN_1707598	SNORD14B	196.158225	169.189425
ILMN_2072391	SNORD31	540.7654125	421.9191375
ILMN_1667288	SNORD73A	178.7578875	161.4384625
ILMN_3248703	SNORD99	319.213025	248.359
ILMN_1685327	SON	183.4407375	163.2802
ILMN_1734645	SPAG4L	125.99125	114.301775
ILMN_1691860	SPRY1	1926.317675	1494.1661
ILMN_2341254	STARD13	107.8647025	119.3140875
ILMN_1750912	STXBP6	164.5014	185.24375
ILMN_2335072	TAF1C	230.0864625	205.6697375
ILMN_1676408	TAGAP	398.4108125	446.5605
ILMN_2333774	TAGAP	720.3453875	391.4004125
ILMN_1739985	TAGAP	823.30565	241.4256125
ILMN_1698179	TAGLN3	117.0457375	135.9438625
ILMN_2060212	TBC1D24	207.3557	177.5095375
ILMN_1684417	TCP10	116.017325	127.3566875
ILMN_1654370	TESK2	260.956	211.586675
ILMN_1709044	TGIF2	849.6235125	684.7035625
ILMN_1786847	TGM3	142.4838125	128.0499875
ILMN_1731048	TLR1	109.31645	120.063875
ILMN_2131336	TMEM194	554.6959875	443.3712875
ILMN_1651535	TMEM217	176.7648875	158.2628875
ILMN_1757129	TMEM88	461.6483	349.762725
ILMN_1736911	TMOD1	118.1209875	146.09275
ILMN_1725014	TMPRSS6	119.4649375	138.757575
ILMN_1719759	TNC	155.7806875	132.505025
ILMN_1731742	TNFRSF13C	150.5800375	129.009325
ILMN_1743100	TNFRSF18	976.6505125	2421.9859625

ILMN_2349633	TNFRSF18	1403.1723375	1880.0266
ILMN_2112256	TNFRSF4	4820.958125	2140.585375
ILMN_1710204	TNFRSF4	928.6155	11140.3355
ILMN_2363392	TNFSF14	6222.198	3418.357625
ILMN_1655414	TNFSF14	5009.827	925.8603125
ILMN_1661343	TNFSF14	1333.8822	4339.690375
ILMN_1746175	TNFSF4	736.301225	1160.5597875
ILMN_2089875	TNFSF4	1816.267575	486.702625
ILMN_1667893	TNS3	135.718975	463.7592125
ILMN_1749218	TOPORS	255.1299375	223.7347
ILMN_3305055	TP63	124.4425125	166.3843875
ILMN_1763104	TRAF4	584.6709875	405.0281125
ILMN_1684943	TRAT1	1225.64015	1953.52375
ILMN_1704972	TRIM5	471.416	373.2538625
ILMN_1775761	TSR1	729.7345875	600.837725
ILMN_2183784	TTC12	121.8393125	140.4326625
ILMN_1672908	TWIST1	124.7775	164.7625375
ILMN_1678841	UBD	136.8637375	281.3033375
ILMN_2241679	UBE2D3	128.6821125	120.6864375
ILMN_1654323	UBE3A	125.92005	119.6828875
ILMN_1750521	UCP3	192.879025	160.4595875
ILMN_1755992	UCP3	162.0526375	139.98565
ILMN_1718136	UQCRHL	5041.99625	6014.628
ILMN_1664537	USP11	423.862175	300.6838625
ILMN_1687284	USP12	295.7662	248.72025
ILMN_1714527	VAMP3	790.535225	656.319525
ILMN_1766955	VCAM1	248.203375	157.6581875
ILMN_2307903	VCAM1	1032.0768125	125.8765
ILMN_1683939	VIPR2	114.8312	122.7872
ILMN_1682996	VWA5A	127.740025	139.0208125
ILMN_1661051	WBP11	132.4729875	119.724475
ILMN_1685763	WDR45L	572.16425	474.8373625
ILMN_1718792	XCL1	1053.031425	2945.11785
ILMN_2224955	XCL1	7781.824125	475.2202
ILMN_1802160	XIRP1	1142.5642875	443.936875
ILMN_1705865	XIRP2	107.4704125	114.8700875
ILMN_1799815	XYLT2	559.72925	766.5865625
ILMN_1731113	ZBTB43	515.8247	417.990675
ILMN_1736954	ZBTB7B	216.3696625	261.0252625
ILMN_1754660	ZCCHC24	202.094975	172.510925
ILMN_1723007	ZCCHC9	1226.031525	1408.7775
ILMN_1715526	ZDHHC21	120.4049125	131.6752875
ILMN_3242993	ZFR2	150.5525125	129.4174625
ILMN_1793621	ZFYVE27	133.7688125	149.7469125
ILMN_1771627	ZMIZ1	2444.37	1715.89925
ILMN_1761965	ZNF200	109.0708125	118.19205

ILMN_1709377	ZNF28	137.0385	127.3357125
ILMN_1700766	ZNF324B	192.3731	167.4530125
ILMN_1692100	ZNF35	149.69075	193.0296125
ILMN_1673804	ZNF426	238.8212	209.9627
ILMN_1734267	ZNF587	115.227025	125.4889125
ILMN_1698048	ZNF619	122.7989	115.8848875
ILMN_1810891	ZNF629	137.3506	125.0395125
ILMN_1670640	ZNF664	160.8478125	145.1453375
ILMN_3251506	ZNF69	129.4988625	141.7017625
ILMN_1734608	ZNF77	226.3165625	194.824775
ILMN_1727574	ZNF827	834.3250875	631.300625

ILMN_GENE: Illumina Gene
