# Supplementary material for: Transcriptomic features of tumour-infiltrating CD4lowCD8high double positive αβ T cells in melanoma
Source: Sci Rep. 2020 Apr 3;10:5900. doi: 10.1038/s41598-020-62664-x (PMC7125144; doi:10.1038/s41598-020-62664-x)
Supplement: Supplementary file 5 — Supplementary information 5. [file 41598_2020_62664_MOESM5_ESM.pdf]

Probe	ILMN_GENE	mean expression in DP	mean expression in CD8
ILMN_1781999	ABCF2	546.074475	615.1121375
ILMN_1716832	ACSF3	136.5403375	150.4636875
ILMN_1679797	ADARB1	1072.2222125	732.5753875
ILMN_2319326	ADARB1	280.3200375	194.244675
ILMN_1657442	ADARB1	242.670075	216.37965
ILMN_1690829	AGER	123.8017875	116.653225
ILMN_1794875	AGPAT9	4764.05225	2805.052125
ILMN_1715532	AKAP12	122.4227375	111.9337375
ILMN_1670870	ALCAM	310.5463125	383.18335
ILMN_1796946	ALLC	106.6048	113.531425
ILMN_1660183	ANKRD13D	116.9001375	126.0616
ILMN_1655868	ANP32C	188.4416625	220.2140125
ILMN_1813840	AOF2	619.5135	728.5491875
ILMN_1731178	ARHGAP28	112.3649875	120.4621625
ILMN_1779256	ARMC2	131.038575	121.96495
ILMN_1700340	ASGR2	118.5828125	126.93515
ILMN_1700625	ATP5S	285.204825	334.397875
ILMN_2298301	BNC1	115.8346875	121.7465125
ILMN_2155172	BRIX1	1144.0847125	1394.63825
ILMN_1676548	BZW2	2599.088125	2989.535625
ILMN_1656463	C11ORF73	203.3215125	227.7316375
ILMN_2180371	C12ORF24	666.7982125	965.7316625
ILMN_1728435	C12ORF45	475.3160375	575.996125
ILMN_2173500	C18ORF51	123.232875	115.8632375
ILMN_1771815	C20ORF43	6465.29825	5285.283875
ILMN_2266005	C21ORF51	314.6141125	549.8450375
ILMN_2356311	C21ORF51	481.1465625	358.3193375
ILMN_1815682	C3ORF37	1426.420375	1150.630575
ILMN_1757137	C4ORF38	113.34315	107.371375
ILMN_1677292	C5ORF30	250.756425	312.601975
ILMN_1659524	C6ORF66	678.481775	835.82275
ILMN_2082699	C8ORFK36	115.8299	123.900925
ILMN_1804339	CAMK1G	135.7508375	122.712425
ILMN_2319544	CAMK2D	154.9016625	178.7578875
ILMN_1785156	CASRL1	113.772025	127.6523625
ILMN_1678061	CASS4	248.3291625	187.8225875
ILMN_1655191	CASZ1	562.0506125	724.583375
ILMN_1686920	CCDC58	693.7295125	878.5679875
ILMN_1745904	CCDC6	1736.755125	2429.603875
ILMN_1769129	CCL19	122.6833125	113.37725
ILMN_1692390	CCNT1	338.5425625	297.7871125
ILMN_1679382	CCT2	3620.764125	4376.7535
ILMN_3239639	CD200R1L	120.1728	111.4048
ILMN_1687825	CD226	860.026625	1098.373075
ILMN_1688959	CD27	210.4026875	610.514675

ILMN_1727284	CD4	453.1392625	111.4364125
ILMN_1659077	CD40LG	6789.513125	2388.150375
ILMN_1666074	CD8B	113.485525	120.99925
ILMN_1802615	CDK6	4081.186625	5578.251875
ILMN_1778917	CDK7	1006.8672875	1169.066475
ILMN_1727815	CFI	148.1957875	164.354475
ILMN_1732750	CHCHD8	262.756375	294.0178375
ILMN_1770548	CLASP2	232.1587	204.9334625
ILMN_1763398	CLEC17A	187.2013875	164.8576
ILMN_1670101	CLEC2D	307.4464	428.4228875
ILMN_1740502	CLEC4GP1	108.310275	114.65485
ILMN_2393994	CSPP1	362.9260875	415.22065
ILMN_1661359	CXORF65	247.1049625	197.689
ILMN_1744458	CYP2W1	116.081875	109.11515
ILMN_3255061	CYTSB	121.6130125	113.307025
ILMN_1730612	DBNDD2	1049.2332	608.199825
ILMN_1739640	DCHS1	135.169875	124.5423625
ILMN_1763129	DCTPP1	2079.851	2649.812125
ILMN_1726839	DCUN1D5	2328.442875	2723.085625
ILMN_1744308	DHX33	866.9904375	1071.6525375
ILMN_1741621	DKFZP434I0714	132.3390875	121.8940875
ILMN_1794692	DNMT3B	258.708175	230.645675
ILMN_1763162	DPH2	252.5981	274.6418
ILMN_1777917	DSCR10	139.1086375	125.311025
ILMN_1791711	DUOXA2	125.026425	118.3780375
ILMN_2275248	ECE2	373.89455	450.3921375
ILMN_1761463	EFHD2	6730.18125	5123.408
ILMN_1797074	EMG1	1890.447	2287.20125
ILMN_1677955	ENTPD6	150.21065	140.623925
ILMN_2166865	ENY2	2846.65525	3249.53375
ILMN_1756989	EPHA8	143.85195	130.382925
ILMN_2104696	ERICH1	878.6670875	963.3490625
ILMN_2055477	EXOSC7	1027.3449375	1218.730625
ILMN_1709233	F5	122.0112375	150.8913125
ILMN_2146761	FABP5	2013.373375	2910.152625
ILMN_1696302	FABP5	295.708775	434.02885
ILMN_3266606	FABP5L2	4410.699	1527.6929125
ILMN_3178258	FABP5L2	1039.170875	5974.0455
ILMN_3238845	FAM165B	537.535925	653.450225
ILMN_1655051	FAM86B1	218.0040625	256.8875375
ILMN_2062687	FARP2	156.9063125	140.79445
ILMN_1768743	FIP1L1	752.1383	830.1787375
ILMN_3185453	FLJ10661	129.69735	141.3337875
ILMN_1705550	FOXD4L2	126.4570625	114.409775
ILMN_1661566	FSTL4	133.168675	122.438225
ILMN_1744914	FUCA2	236.0935125	180.620925

ILMN_2280548	FYB	106.771275	114.4816875
ILMN_1709032	FYCO1	896.0827875	720.16865
ILMN_1679476	GART	1592.47725	800.9108875
ILMN_1793220	GART	696.881025	1774.88
ILMN_1797482	GCDH	220.000775	522.9815875
ILMN_2296843	GCDH	414.4951125	264.2001
ILMN_1707484	GEMIN6	758.0822375	960.060925
ILMN_1769388	GJB2	135.3812125	171.9277875
ILMN_2226223	GJB6	120.7590125	140.119475
ILMN_1718671	GJB6	128.669275	157.1667375
ILMN_1806754	GLDC	282.2840375	854.8050875
ILMN_1736757	GNPTAB	991.5712875	1312.521
ILMN_1730816	GPR162	128.0344625	148.06015
ILMN_1725271	GPR3	156.189125	144.9764125
ILMN_3242603	GTF2H2B	516.9658375	576.6771375
ILMN_1725666	GTF2H3	291.780225	311.4122
ILMN_1696330	GUF1	251.9959875	285.6177625
ILMN_1693826	HAVCR2	4491.687875	6880.74625
ILMN_1704089	HPN	113.1919375	108.18683375
ILMN_1863284	HS,130916	544.1257	621.0486
ILMN_1828747	HS,147255	115.4578875	128.1549125
ILMN_1849907	HS,195035	129.98635	123.867375
ILMN_1821915	HS,353831	122.6687375	129.3806125
ILMN_1862684	HS,373429	637.4927625	933.234925
ILMN_1819494	HS,406106	256.384225	337.6049875
ILMN_1868808	HS,434799	115.369	158.696975
ILMN_1821681	HS,529324	131.876125	140.806975
ILMN_1869823	HS,543130	131.8411625	140.0752125
ILMN_1888935	HS,549668	123.1046625	134.2802625
ILMN_1912197	HS,567480	125.4754125	196.1495875
ILMN_1855797	HS,568093	154.2935875	171.9240625
ILMN_1890362	HS,570751	153.1664125	173.5394625
ILMN_1848214	HS,571599	129.5084875	139.4365875
ILMN_1889135	HS,572124	128.867075	121.7592125
ILMN_1771921	HSCB	219.6125875	246.690475
ILMN_1803775	HSPE1	2589.469375	3525.34025
ILMN_1696654	IFIT5	161.0033625	135.1091125
ILMN_1774983	IL17A	123.13565	134.1094375
ILMN_1767523	IL17RB	7183.2896875	3119.9035
ILMN_2407799	IL24	226.1770375	190.247825
ILMN_1753758	IL27	113.4241125	105.9127
ILMN_1667575	IL31RA	121.9088	115.419625
ILMN_1669174	IL4	10095.381	142.273325
ILMN_2389080	IL4	182.2643125	6093.39225
ILMN_1727248	INF2	338.36855	274.9224875
ILMN_1660844	INTS4	624.5129875	544.589775

ILMN_1811468	IRX3	5444.00175	2294.831375
ILMN_1711650	KCNE1L	152.3193125	134.67485
ILMN_1780334	KCNJ2	208.4257375	156.087925
ILMN_1740508	KCNMA1	114.5354	107.2019625
ILMN_1693233	KIAA0513	233.814625	179.1771125
ILMN_1782222	KIAA1033	122.301325	132.063275
ILMN_1752273	KIAA1143	495.5788875	598.1752625
ILMN_1794528	KIAA1751	125.129675	113.0530125
ILMN_1772786	KIF16B	129.6914	120.093075
ILMN_1703314	KLHL36	1296.943375	1104.496575
ILMN_2189222	KLHL8	580.86585	670.9714375
ILMN_2384139	KLRC1	117.1271	441.0732875
ILMN_1752647	KLRC1	128.5708125	313.0066
ILMN_2384140	KLRC1	131.9296875	224.6144375
ILMN_2059357	KLRC2	118.7008	240.3815875
ILMN_2386790	KLRC3	129.4593125	260.0630375
ILMN_1797988	KLRD1	366.7452875	792.1636625
ILMN_1703949	KPNB1	4285.9145	4829.42275
ILMN_1794729	KRT20	116.5591375	124.6523625
ILMN_1813338	LAG3	4900.2705375	8638.966625
ILMN_2347888	LARP4	488.35925	575.459325
ILMN_1651328	LAT1-3TM	246.4572125	309.507425
ILMN_2377109	LCK	653.97465	522.1208875
ILMN_2412214	LGALS9	175.9085875	143.1300625
ILMN_1665011	LHX3	111.4308875	123.8980375
ILMN_1738725	LIF	758.4126375	422.7844875
ILMN_3262025	LOC100127993	7251.109125	8085.7555
ILMN_3268914	LOC100128410	8273.6025	9901.028125
ILMN_3188196	LOC100128862	121.782425	132.9273125
ILMN_3189463	LOC100129054	113.7131875	121.4208
ILMN_3188174	LOC100129067	145.6984125	156.370825
ILMN_3178544	LOC100129436	113.7917625	120.5027875
ILMN_3259185	LOC100129630	144.6052125	131.9551375
ILMN_3178320	LOC100130288	117.2889625	127.0217625
ILMN_3265391	LOC100130413	148.6963125	140.39275
ILMN_3258321	LOC100130932	691.8457625	875.5185625
ILMN_3215992	LOC100131320	111.5887875	118.09085
ILMN_3296811	LOC100131416	114.0026625	121.5770875
ILMN_3215392	LOC100131514	112.5388625	120.5565625
ILMN_3202653	LOC100132493	208.477275	231.6728125
ILMN_3203444	LOC100132535	137.4880625	159.9787875
ILMN_3275936	LOC100133277	225.5982875	283.01955
ILMN_3291287	LOC100133284	118.87825	125.9745875
ILMN_3203189	LOC100133591	324.6024625	290.1535375
ILMN_1677852	LOC284232	116.5699	127.2694125
ILMN_3204890	LOC345041	188.594825	217.4819625

ILMN_3218851	LOC402116	114.2138875	122.639775
ILMN_1706434	LOC440359	546.1680875	826.024775
ILMN_1655842	LOC442597	178.046175	219.6740875
ILMN_1676613	LOC642597	111.6486375	121.764525
ILMN_1688246	LOC642852	613.3163125	485.405475
ILMN_3210741	LOC642956	1105.7627125	1604.8732875
ILMN_1735758	LOC643062	126.329925	117.343125
ILMN_1657728	LOC643831	163.5770125	186.59005
ILMN_1723213	LOC644916	112.081075	122.454625
ILMN_1719111	LOC645200	116.121575	108.5398125
ILMN_1746634	LOC645454	110.7037875	117.5292875
ILMN_1666706	LOC645676	232.858875	260.588525
ILMN_1691405	LOC645850	121.6853375	112.13425
ILMN_1672624	LOC646110	117.9216125	109.4688625
ILMN_1667933	LOC646428	110.8037125	118.440925
ILMN_1696526	LOC646448	109.22295	119.11
ILMN_1780669	LOC647513	121.6740125	111.4983125
ILMN_1700880	LOC648399	151.3563625	167.8322125
ILMN_1694313	LOC649139	124.432725	116.7930125
ILMN_1654493	LOC649169	262.3705375	311.6399
ILMN_1683664	LOC650369	3648.25325	4352.583375
ILMN_1803231	LOC650543	118.318025	128.9808875
ILMN_1783156	LOC650832	1724.120375	1321.6876
ILMN_1772906	LOC652086	122.8814	112.9976
ILMN_1696397	LOC652833	119.952625	129.193525
ILMN_1683115	LOC652843	119.4093125	132.443475
ILMN_1749336	LOC652848	124.2272125	116.578425
ILMN_1764944	LOC653562	127.01865	119.100825
ILMN_3204117	LOC728115	304.831975	360.045775
ILMN_3285231	LOC728148	119.3678625	142.7594
ILMN_3301813	LOC728470	134.4428125	122.0785
ILMN_3304519	LOC728576	18917.4025	20784.07875
ILMN_3243807	LOC728933	115.8670375	111.151175
ILMN_3224126	LOC729222	144.955925	126.9178875
ILMN_3298549	LOC729251	121.8936125	109.8609625
ILMN_1651351	LOC729444	112.678975	121.7796375
ILMN_3228320	LOC729590	119.12825	109.3235625
ILMN_3226392	LOC729608	2247.083	2626.1065
ILMN_3304584	LOC729739	121.32065	130.21095
ILMN_3302484	LOC729774	389.5021625	460.42975
ILMN_3306482	LOC730107	369.477025	494.9788625
ILMN_1844813	LOC731670	112.0030875	119.1547125
ILMN_1723443	LRP2	124.7256125	114.5224
ILMN_2131756	LRRC40	117.6986125	123.5685875
ILMN_1792927	LTBP3	122.8187875	115.9042125
ILMN_1799871	LTK	123.19545	113.1947

ILMN_1663131	LYST	188.4673375	422.9119375
ILMN_1680139	MAFF	264.9656	194.6782625
ILMN_1661417	MAGED2	125.830025	138.3467125
ILMN_1657478	MAGEL2	366.9208125	268.0502
ILMN_1759341	MAN2B1	348.1433	298.0708125
ILMN_1790534	MAP2K3	2152.13025	1678.009
ILMN_1714162	MARLIN1	169.1195625	192.5519
ILMN_1764082	MBOAT1	429.9389	317.58695
ILMN_1769264	MCCC2	163.0164625	176.084225
ILMN_3248910	MIR155HG	6132.694625	7647.618375
ILMN_3310326	MIR221	327.4981125	392.9595875
ILMN_1796316	MMP9	252.678125	170.112375
ILMN_1794589	MRGPRX2	126.2104625	116.7599625
ILMN_2076658	MRPL1	808.1583	950.4123125
ILMN_2103720	MRPL15	921.798475	1141.0150875
ILMN_2348050	MRPL21	1487.393	1758.15825
ILMN_2230592	MRPL3	133.84605	144.9250875
ILMN_1661039	MRPL30	151.4707875	166.88165
ILMN_2356895	MRPL42	568.2228875	689.8373
ILMN_1804851	MRPS17	2102.64225	2519.716875
ILMN_1711414	MRPS27	740.1130375	822.970375
ILMN_2308849	MYADM	6988.457875	4806.96075
ILMN_1711894	MYB	720.8530125	956.2975
ILMN_1679891	NAF1	225.6475625	269.8769625
ILMN_1780291	NFAT5	437.2167125	382.1605625
ILMN_1717313	NFKBIE	547.412375	442.083825
ILMN_1719695	NFKBIZ	2481.52	1879.632625
ILMN_1697614	NHP2L1	667.7166625	780.143825
ILMN_1815086	NINJ1	6671.591625	5561.010125
ILMN_1713638	NIPAL4	122.743125	134.3905875
ILMN_1750052	NOP14	844.1636625	1005.4336125
ILMN_1748476	NOP58	4749.766625	5868.03675
ILMN_2410145	NR4A1	158.034375	125.849225
ILMN_2408566	NR4A1	170.667975	136.190925
ILMN_1774281	NT5DC3	121.742125	130.958425
ILMN_1658247	OAS1	213.61135	173.3330875
ILMN_1758129	OR6F1	116.2108125	110.6707875
ILMN_1773278	OR8D4	115.1343	110.453625
ILMN_1709091	OXGR1	121.9367875	113.891775
ILMN_1744713	PARK7	7985.6615	9165.712125
ILMN_1810100	PBX3	336.6899125	263.970475
ILMN_1665679	PEX10	112.0400375	119.4223
ILMN_1790533	PHACTR2	746.6902	495.70225
ILMN_1775901	PHF5A	625.1172375	819.5880875
ILMN_1666819	PHLDB1	213.1802125	184.0668625
ILMN_1758111	PIBF1	166.3757625	187.82245

ILMN_1715273	PICALM	2363.333	1817.9715
ILMN_2182647	PINX1	286.27205	321.66275
ILMN_1790309	PINX1	453.7997625	580.2410125
ILMN_1735693	PIWIL3	104.95841	114.1555375
ILMN_1691508	PLAUR	165.58435	137.2867
ILMN_1785265	PLS3	123.506625	161.944175
ILMN_2041788	PLS3	112.07755	206.866125
ILMN_1689525	PMAIP1	192.174875	217.4401125
ILMN_2098446	PMAIP1	516.162175	675.1203875
ILMN_1756445	PMF1	447.8152875	525.9251875
ILMN_1768273	POP1	426.451875	465.03065
ILMN_1798459	PPAN	987.6323	1168.0958875
ILMN_2294784	PRDM1	848.2657375	645.0980625
ILMN_1797776	PRSS23	155.6725625	199.26775
ILMN_2344130	PSMD4	1864.820875	2064.768625
ILMN_1747023	PTGIR	152.879	129.9924375
ILMN_1721046	PTMS	867.365525	1294.6846625
ILMN_1769779	PTP4A3	367.1384375	203.4652625
ILMN_2359710	PTP4A3	364.5910625	195.6415875
ILMN_1779353	PUS7	825.97105	985.7057125
ILMN_2221006	RAD21	1180.554225	1397.77595
ILMN_1651902	RASL11A	121.723175	134.26325
ILMN_1740716	RBM26	805.982025	914.11315
ILMN_1793033	RBM28	410.022925	453.1400625
ILMN_2169383	REG4	137.0624125	405.296475
ILMN_1661002	RFWD2	1089.929	941.3754125
ILMN_1770733	RIC8A	2163.802125	1529.873375
ILMN_1687922	RP9	764.3657625	924.3325
ILMN_1664167	RPF2	2653.64325	3124.493375
ILMN_2399893	RPS24	1236.1758625	1588.186325
ILMN_3235477	RRP1	431.53025	513.2409875
ILMN_1689972	RRP9	259.1259	298.97575
ILMN_1756928	RTN1	105.856275	114.362225
ILMN_1687315	RXRA	732.051575	349.614475
ILMN_1810857	S100A5	130.3487125	120.4697
ILMN_2399208	SCAMP3	217.55655	244.4213
ILMN_1781656	SCGB1D1	112.6163625	120.710975
ILMN_2363591	SDCBP	1767.809025	1236.3559
ILMN_2277246	SDCBP2	121.6141375	110.08915
ILMN_1676067	SEMA3G	107.0005	116.3046375
ILMN_2233878	SERF1B	320.703025	360.6852125
ILMN_1744381	SERPINE1	259.8637125	208.2204125
ILMN_1720513	SETBP1	185.0626375	262.0766
ILMN_1751075	SETD4	238.0386125	265.90105
ILMN_1795976	SFXN2	174.302375	235.8015125
ILMN_3249594	SGK196	125.7911	115.2136875

ILMN_1792933	SLC10A6	118.5314125	111.7119375
ILMN_1785405	SLC17A9	147.213075	177.4708
ILMN_1781231	SLC25A38	567.520225	647.0346875
ILMN_2258471	SLC30A5	129.059475	146.4302125
ILMN_1789001	SLC35B2	700.04025	875.0830375
ILMN_2330307	SLC43A3	186.278825	314.3947125
ILMN_1658407	SLC43A3	234.9404375	441.550425
ILMN_1655740	SNAI2	119.277775	126.895825
ILMN_1683273	SNAPC5	195.2155125	221.3478625
ILMN_1772845	SNRNP35	357.5517	307.1135125
ILMN_1736093	SNX33	217.14715	175.9598875
ILMN_1728714	SSSCA1	355.6278625	428.1416875
ILMN_1799600	STARD8	208.2712875	161.2999625
ILMN_1750912	STXBP6	164.5014	137.08275
ILMN_2059549	SYK	113.10155	107.2457875
ILMN_1764087	SYPL1	940.255825	673.886425
ILMN_1787081	TADA2A	202.1870875	220.0502
ILMN_1771949	TAF4B	135.588475	145.927525
ILMN_2292387	TANK	278.7298875	328.0089875
ILMN_1688098	TBC1D4	380.8013	723.6355875
ILMN_1744795	TBL1X	3286.946375	2130.645
ILMN_1706839	TCERG1	422.9682625	498.3699375
ILMN_1684417	TCP10	116.017325	127.27535
ILMN_1653529	TEX10	446.959525	550.1304625
ILMN_1814657	TFAP4	150.4829125	166.679375
ILMN_2067708	TFB2M	2262.335875	2714.500125
ILMN_1782688	THNSL1	142.5418	165.6422875
ILMN_1750678	TIMD4	187.3600625	416.20185
ILMN_1706419	TM6SF2	121.235525	128.7928125
ILMN_1794677	TMC6	1605.748375	1244.835875
ILMN_1667716	TMEM101	604.2959	690.9420125
ILMN_1756238	TMEM217	356.92925	289.1355875
ILMN_1728202	TMEM22	284.3303125	219.1552625
ILMN_2394523	TMEM231	123.991725	132.543975
ILMN_2414325	TNFAIP8	1783.823875	2260.349
ILMN_2349633	TNFRSF18	1403.1723375	853.9839125
ILMN_1746175	TNFSF4	736.301225	2623.86475
ILMN_2089875	TNFSF4	1816.267575	1032.89775
ILMN_1761778	TNFSF8	1546.5949	846.279175
ILMN_1707591	TNIP3	1602.787375	2622.035625
ILMN_1667893	TNS3	135.718975	488.2152125
ILMN_1704576	TP53AIP1	112.4059125	124.3733375
ILMN_2094718	TPT1	24526.41125	23076.54
ILMN_1694817	TRH	106.924125	113.8623875
ILMN_1680993	TRIM10	115.478625	123.036175
ILMN_1764098	TRNT1	150.3962375	164.09565

ILMN_1655924	TRNT1	367.7509125	428.420925
ILMN_1746393	TSEN2	557.285575	653.3695875
ILMN_1663113	TTLL12	510.09385	654.2704375
ILMN_1746846	TTLL4	218.4945875	235.8945125
ILMN_1653712	UAP1L1	144.5518875	164.5055125
ILMN_1768662	UCK2	650.4554625	761.9075625
ILMN_1714527	VAMP3	790.535225	609.5617625
ILMN_2325763	VCAM1	160.5983625	1988.1085875
ILMN_1766955	VCAM1	248.203375	434.775025
ILMN_2307903	VCAM1	1032.0768125	218.4731
ILMN_2045729	WDR12	1403.638125	1663.542625
ILMN_1789775	WDR74	1141.6463625	1389.660975
ILMN_3235472	WDYHV1	246.1613	277.7600375
ILMN_1706612	WFDC2	110.3114875	119.6734375
ILMN_1679881	WRN	539.57475	610.6687125
ILMN_1759495	XPO5	731.441975	869.2970375
ILMN_1732981	ZBTB32	1407.80255	2495.999375
ILMN_2075847	ZC3H10	222.8152125	239.18845
ILMN_1793621	ZFYVE27	133.7688125	145.214075
ILMN_1775138	ZIM2	199.0836375	170.2409875
ILMN_1695847	ZKSCAN5	173.8131375	156.6375
ILMN_1798533	ZNF22	877.6591625	1251.410125
ILMN_2061310	ZNF280C	270.4831125	320.3470875
ILMN_1710873	ZNF330	629.372475	737.9168375
ILMN_1786722	ZNF385A	124.9977	115.936725
ILMN_1670640	ZNF664	160.8478125	148.164675
ILMN_1738124	ZNF772	134.234675	120.667075
ILMN_1812478	ZNHIT3	3086.460625	576.872825
ILMN_1750044	ZNHIT3	471.37625	3618.8195
ILMN_1675377	ZSCAN2	186.749125	203.0078625
ILMN_1653163	ZSCAN2	176.8938875	201.4655875

ILMN_GENE: Illumina Gene
