# Supplementary material for: Transcriptomic features of tumour-infiltrating CD4lowCD8high double positive αβ T cells in melanoma
Source: Sci Rep. 2020 Apr 3;10:5900. doi: 10.1038/s41598-020-62664-x (PMC7125144; doi:10.1038/s41598-020-62664-x)
Supplement: Supplementary file 6 — Supplementary information 6. [file 41598_2020_62664_MOESM6_ESM.pdf]

Gene	Forward	Reverse
CTNNB1 (b-catenin)	AGGTCTGAGGAGCAGCTTCA	TTCAAATACCCTCAGGGGAACA
LEF1	GACCTAATGCACGTGAAGCC	CACTCAGCAACGACATTCGC
TCF1	CCCCCGCTGCACAAGG	CAGAGGCCTGTGAACTTGCT
thPOK	CCCTGTCTGCCACAAGATCA	CAGCTTGTCGTTCCTGGTGA
RUNX3	CCCCCTGAAGGGCTGAAAAT	CTGGCCACCTGGTTCTTCAT
TBX21 (T-bet)	PPH00396A-200 (Qiagen)	
EOMES	PPH12647A-200 (Qiagen)	
